# Inhibition of JNK/c-Jun-ATF2 Overcomes Cisplatin Resistance in Liver Cancer through down-Regulating Galectin-1

**DOI:** 10.7150/ijbs.79163

**Published:** 2023-04-25

**Authors:** Fan Yang, Mengzhu Li, Duo Xu, Zebo Jiang, Hailong Jiang, Yitai Xiao, Chaoming Mei, Meilin Yang, Congmin Chen, Bin Zhou, Bailiang He, Hong Shan, Pengfei Pang, Dan Li

**Affiliations:** 1 Department of Nuclear Medicine, The Fifth Affiliated Hospital, Sun Yat-sen University, Zhuhai, Guangdong Province 519000, China; 2 Guangdong Provincial Engineering Research Center of Molecular Imaging, The Fifth Affiliated Hospital, Sun Yat-sen University, Zhuhai, Guangdong Province 519000, China; 3 Center for Interventional Medicine, The Fifth Affiliated Hospital, Sun Yat-sen University, Zhuhai, Guangdong Province 519000, China; 4 Zhongshan School of Medicine, Sun Yat-sen University, Guangzhou, Guangdong Province 510080, China

**Keywords:** *In vivo* bioluminescence imaging, Cisplatin, Resistance, JNK, Liver cancer.

## Abstract

Due to drug resistance, the clinical response to cisplatin (CDDP) from patients with liver cancer is unsatisfactory. The alleviation or overcoming of CDDP resistance is an urgent problem to be solved in clinics. Tumor cells rapidly change signal pathways to mediate drug resistance under drug exposure. Here, multiple phosphor-kinase assays were performed and c-Jun N-terminal kinase (JNK) was activated in liver cancer cells treated with CDDP. The high activity of the JNK promotes poor progression and mediates cisplatin resistance in liver cancer, leading to a poor prognosis of liver cancer. Mechanistically, the highly activated JNK phosphorylated c-Jun and ATF2 formed a heterodimer to upregulate the expression of Galectin-1, leading to promoting cisplatin resistance in liver cancer. Importantly, we simulated the clinical evolution of drug resistance in liver cancer by continuous CDDP administration* in vivo*. *In vivo* bioluminescence imaging showed the activity of JNK gradually increased during this process. Moreover, the inhibition of JNK activity by small molecular or genetic inhibitors enhanced DNA damage and overcame CDDP resistance *in vitro* and* in vivo.* Collectively, our results underline that the high activity of JNK/c-Jun-ATF2/Galectin-1 mediates cisplatin resistance in liver cancer and provides an optional scheme for dynamic monitoring of molecular activity *in vivo*.

## Introduction

Liver cancer is one of the most frequent human solid tumors with a poor prognosis and ranks as the third-leading cause of cancer death globally 1. Chemotherapy, including cisplatin (CDDP), remains a potent treatment for patients with late-stage liver cancer 2, 3. Along with long-term chemotherapy, most patients have a less efficient response and ultimately develop resistance to the CDDP unfortunately 4. Therefore, molecular mechanisms of CDDP therapeutic resistance still need further investigation. Many classic mechanisms of chemoresistance have been widely researched from a genetic perspective, such as the abnormality of DNA damage repair (DDR) related genes, increased detoxification activity, the repressed chromatin state and drug efflux promoted by the high expression of multidrug resistance-associated protein 2 5. However, various evidence shows cell signal changes are emerging as a vital contributor to drug resistance without generating new genetic modifications 6-8.

Alterations affecting tumor sensitivity to drugs in signaling pathways have attracted widespread attention 9. Protein kinases are essential molecules that rewrite signal networks under the therapeutic pressures involving tumorigenesis and tumor progression 10. The c-Jun N-terminal kinase (JNK), belonging to the MAPK family (also known as stress-activated protein kinase SAPK), is activated in response to various stimuli, such as infection, oxidative stress, cytotoxic drugs, cytoskeletal changes and DNA damage 11. The activation of JNK generally regulates gene expression by phosphorylating downstream transcription factors, such as c-Jun and activating transcription factor 2 (ATF2), which are oncogenic 11-13. It was reported that the VASH2/c-Jun/RRM2 axis mediates pancreatic cancer resistance to gemcitabine 14. And some evidence also demonstrated that the Smad1/ATF2/p57Kip2 signal triggers doxorubicin resistance in colon cancer 15. For note, the function of JNK activation induced by drugs or small molecules has two sides. In colon cancer, putrescine triggers 5-fluorouracil resistance by activating JNK 16. In contrast, the activation of JNK by doxorubicin decreased its cytotoxicity in breast cancer 17. Besides, the activation of c-Jun and ATF2 by JNK constructs heterodimer to promote cancer cell proliferation 18. Therefore, the role of JNK and the corresponding downstream of this signal in liver cancer response to cisplatin therapy need to be further explored. Moreover, the real-time monitoring of malignant kinase activity enables us to assess therapeutic response earlier and more precisely, which necessitates the development of a strategy to trace the activity of JNK signal* in vivo* 6.

In this study, we observed that CDDP resistance of liver cancer is due to CDDP-induced activation of JNK/c-Jun-ATF2/Galectin-1. JNK promotes c-Jun-ATF2 to transcriptionally upregulate Galectin-1, resulting in CDDP resistance. The results herein suggest that the high activation of JNK signal was associated with the acquisition of CDDP resistance in liver cancer. Furthermore, *in vivo* bioluminescence imaging (BLI) showed that the activity of JNK gradually increased during the evolution of CDDP resistance. Taken together, this work revealed that JNK/c-Jun-ATF2/Galectin-1 is a valuable target to overcome CDDP resistance in liver cancer and proposed a feasible approach to trace JNK activity *in vivo*.

## Materials and methods

### Reagents and antibodies

The reagents used in this study are listed in Table S1. Antibodies used here are listed in Table S2.

### Cell lines and cultures

The human liver cancer cell lines Hep 3B and SK-Hep 1 were obtained from ATCC. Hep 3B and SK-Hep 1 were respectively grown in DMEM and RPMI-1640 supplemented with 10% fetal bovine serum and 1% penicillin-streptomycin. CDDP and SP600125 were melted and stored according to the manufacturer's instructions.

### Generation of cisplatin-resistant cell line

The CDDP-resistant cell line (Hep 3B/DR) was constructed by CDDP low-dose continuous stimulation. The specific steps are as follows. Cells were treated with CDDP at different concentrations (10, 5, 2.5 and 1.25 μM) for 24 h. Then, they were cultured in the drug-free medium for another three days. Since only a small fraction of cells survived in the 1.25 μM group, this group was selected for the next induction. The cells were continuously cultured in 1.25 μM CDDP medium until they could proliferate stably. The medium was replaced every three days, and the cells were passaged when they grew to 90% confluence. This incubation process was repeated until cells could grow stably in 2.5 μM and 3.3 μM CDDP stepwise. At last, Hep 3B/DR cells were normally cultured in the medium containing 3.3 μM CDDP.

### Human Phospho-Kinase Array

Hep 3B cells were treated with CDDP (20 μM, 18 h) or DMSO. The total protein content of cell lysates was quantified by BCA assay. Profiling of phosphoproteins was performed by equal amounts of protein (300 μg) from cell lysates using Human Phospho-Kinase Array according to the manufacturer's instructions. In this array, 37 captured antibodies were spotted in duplicate on nitrocellulose membranes. Chemiluminescent images were obtained using ChemiDoc/XPS+ (BIO-RAD). The background signal correction and the average signal of duplicate spots were analyzed by ImageJ software. The values were normalized to the positive control spots on each membrane.

### Western blot

The detailed procedure was performed as previously 21. Briefly, cells were lysed with RIPA lysis buffer containing protease inhibitor cocktail. The cells lysates were centrifuged at 13000 rpm and the concentration of supernatant was quantified by BCA protein quantification kit. The protein was boiled with loading buffer for 5 min at 100°C. Equal amounts of proteins from the different treatment groups were fractionated by SDS-PAGE and electro transferred to PVDF membranes. After being blocked, the membranes were incubated with primary antibodies at 4℃ overnight. They were then incubated with the horseradish peroxidase-conjugated secondary antibodies. After being incubated in ECL detection reagent, signals were visualized by ChemiDoc/XPS+ system. Primary antibodies for JNK, c-Jun, ATF2, p-JNK (Thr183/Tyr185), p-c-Jun (Ser73), p-ATF2 (Thr69/71), γ-H2AX (Ser139) and Galectin-1/LGALS1 used here are from Cell Signaling Technology, while GAPDH is from Proteintech. More information on antibodies used here is listed in Table S2.

### Co-immunoprecipitation (Co-IP) assays

Cellular extracts were obtained by incubating cells with RIPA lysis buffer. Supernatants were obtained by centrifugation at 12000 rpm for 15 min at 4°C. For immunoprecipitation, 600 μg of protein was incubated with primary antibody or IgG according to the recommended proportion overnight at 4°C with rotation. Dynabeads® Protein A was then added and incubated for 1 h at room temperature. The complexes were washed five times with RIPA lysis buffer. The protein was eluted with 1×SDS loading buffer and boiled for 15 min followed by western blot analyses.

### ATF-decoy and plasmids

ATF-decoy and ATF-mut-decoy were synthesized by the company (Sangon Biotech) and sequence information was displayed in Table S3. IGEbio was commissioned to construct plasmids pGPU6-Galectin-1-shRNA, pGL4.14-Galectin-1-promoter-Fluc (Gal-1-WT-Fluc) and pGL-4.14-Galectin-1-promoter (ATF-mutant)-Fluc (Gal-1-mut-Fluc). The human Galectin-1 promoter sequence (-3000 to +67) was acquired from NCBI (NM_002305).

### Lentiviral constructs and transductions

Genechem (Shanghai Genechem Co., Ltd.) was commissioned to perform lentivirus (2×ATF-Luc2-TK-hRluc or shGalectin-1) package. 72 h post-infection, SK-Hep 1 cells were cultured in media containing puromycin, and the survival cells were named SK-ATF-Luc or SK-Hep 1^shGalectin-1^ used for animal experiments. Sequence of 2×ATF was listed in Table S3.

### *In vitro* bioluminescence imaging (BLI)

The SK-ATF-Luc cells were used to assess the activity of ATF element after different treatments *in vitro*. The signal of Luc2 and hRLuc were acquired and measured in the same well using IVIS (Lumina Ⅲ, PerkinElmer). The cultured cells were treated with ViviRen (a final concentration of 60 μM) and hRLuc signals were acquired 3 min later. After another 30 min, cells were treated with D-luciferin (150 μg/mL) and Luc2 signals were acquired 5 min later. The relative activity of the ATF element was a ratio of the total photon from Luc2 to hRluc. hRluc was used as an endogenous control of cell numbers.

### Dual-luciferase reporter assay

Gal-1-WT-Fluc or Gal-1-mut-Fluc and pLR-Renilla plasmid were co-transfected to cells at ratio of 9:1 before drug treatment. To detect the effect of declined c-Jun-ATF2 on the activity of Galectin-1 promoter, cells were also co-transfected with the two plasmids above and ATF-decoy or mut-ATF-decoy. Dual-Luciferase® Reporter Assay was used to detect the activity of firefly and renilla luciferase.

### Immunofluorescence (IF)

The detailed procedure was performed as previously 22. Briefly, after drug treatment, the cells were fixed in 4% paraformaldehyde at room temperature for 15 min. After being permeabilized, cells were blocked with 5% BSA in PBS. Subsequently, the cells were incubated with the primary γ-H2AX antibody at 4℃ overnight. After washing with PBS, they were incubated with Dylight 549-conjugated goat anti-rabbit IgG. The cells were stained with DAPI before being captured with Laser Scanning Microscopes 880 (ZEISS).

### Alkaline comet experiment

Liver cancer cells were incubated with 20 μM CDDP alone, or in combination with 20 μM SP600125 for 18 h. For the functional experiment of Galectin-1, cells were transfected with shGalectin-1 before incubating in CDDP. Cells were then collected and used for the Alkaline comet experiment according to the protocol from reagent providers. In short, cells were scraped and resuspended in PBS (1 × 10^5^ cells/mL). The cell resuspension solution was mixed with LMAgarose (previously equilibrated to 37℃) in a volume of 1:10. Next, it was spread on the CometSlideTM glass slide. They were allowed solidification and incubation in lysis solution. The condition of alkaline electrophoresis was 21 V for about 30 min in Alkaline Electrophoresis Solution at 4 ℃. After being rinsed in water, incubated with 70% ethanol and dried in an incubator at 45 ℃, the glass slides were further stained with SYBR Green I diluted at 1:10000 in Tris-EDTA. Images were obtained by an upright fluorescence microscope (OLYMPUS, BX53). CASP Comet Assay Software Project was used for quantitative analysis.

### Bioinformatic analysis of target gene of c-Jun-ATF2

ENCODE Transcription Factor Targets dataset and TRANSFAC Curated Transcription Factor Targets dataset were downloaded from the website (https://maayanlab.cloud/-Harmonizome/). Predicted targets of c-Jun were acquired from ENCODE, TRANSFAC and hTFtarget (http://bioinfo.life.hust.edu.cn/hTFtarget). Predicted targets of ATF2 were acquired from ENCODE. Tumor RNA-seq data of liver cancer patients and paired normal data were obtained from the Genomic Data Commons data portal. The overlapped genes analysis was performed in http://bioinformatics.psb.ugent.be/webtools/Venn/.

### RNA isolation and qPCR analysis

RNA was obtained from cultured cells by Total RNA Kit Ⅰ according to the protocol from the manufacturer. About 1 μg RNA was subsequently used to generate cDNA with PrimeScriptTM RT reagent Kit. TB GreenR Premix Ex TaqTM Ⅱ and qPCR analysis system (BIO-RAD, CFX Maestro) were used to carry out qPCR according to the manufacturer's instructions. The relative expression was analyzed according to the ΔΔCT relative quantification method and normalized to the expression of *ACTB*. Expression measurements of mRNA were performed in triplicate. The primers are listed in Table S3.

### Chromatin Immunoprecipitation assay (ChIP)

ChIP assay was performed according to the manufacturer's protocol. Briefly, 1 × 10^7^ cultured SK-Hep 1 cells were cross-linked in 1% formaldehyde for 10 min and quenched in 1×glycine solution for 5 min at room temperature. The fragmented chromatin was obtained after being treated with nuclease (1.5 μL) and sonication. Chromatin immunoprecipitation was performed with c-Jun antibody, ATF2 antibody and normal rabbit IgG. The sample was reversed cross-linking and DNA purification, and the DNA was quantified by qPCR with primers for c-Jun: ATF2 binding sites in the *Galectin-1* promoter (-1140 to -1011). Fold enrichment was expressed as a percent of the total input. And the products were used for 2% agarose electrophoresis. The primers are listed in Table S3.

### *In vivo* bioluminescence imaging

*In vivo*, BLI signals were quantified using IVIS (Lumina Ⅲ, PerkinElmer) equipped with a charged coupled device (CCD) camera. Mice received tail vein injection of hRluc substrate ViviRen™ (0.295 mM, 75 μL) 7 min before imaging. After 2 h, 150 mg/kg Luc2 substrate D-luciferin was injected intraperitoneally 15 min before imaging. Mice were anesthetized with vaporized isoflurane. Photons were acquired from “region of interest” (ROI) that covered an equal area of the subcutaneous tumor to quantify signal. The relative activity of the ATF element was a ratio of the total photon from Luc2 to hRluc. hRluc was used as an endogenous control of cell numbers.

### Animals and tumor models

Approximately 5 × 10^6^ SK-ATF-Luc or SK-Hep 1^shGalectin-1^ cells were transplanted subcutaneously on the back of female nude mice aged 4 weeks till the tumors' volume reached 100 mm^3^. To trace the dynamic change of JNK during CDDP resistant evolution, the SK-ATF-Luc* xenograft* models were administered CDDP intraperitoneally at 5 mg/kg or equal PBS every 3 day for a total of 28 days. The luciferase activity of tumors was detected by BLI at different time points. And the SK-Hep 1^shGalectin-1^ *xenograft* models were treated with CDDP (5 mg/kg) or equal PBS for function experiment of Galectin-1 knockdown.

The animal drug-resistant model was constructed by two rounds of drug administration referring to the construction methods in other study 23. Briefly, 5 × 10^6^ SK-ATF-Luc cells were injected subcutaneously in nude mice. When the volume of tumor reached 100 mm^3^, mice were treated with CDDP (intraperitoneal, 10 mg/kg each week) for 3 weeks. The tumor tissues with poor CDDP treatment results were retransplanted to new recipients and animals received CDDP treatment for another course. At the end of the second course, the procedure of retransplant was performed as previous step. These animals were recognized as CDDP-resistant models. CDDP-resistant mice were used for combination treatment analysis to determine the effect of JNK inhibition on CDDP efficacy. BLI was also used to monitor luciferase activity.

### Immunohistochemistry (IHC)

The detailed procedure was performed as previously 22. The formalin-fixed and paraffin-embedded sections were baked at 60℃ for 2 h. The slides were dewaxed and hydrated by xylene and graded alcohol before undergoing heat-induced epitope retrieval at 110℃. Next, 3% H_2_O_2_ was used to block the endogenous peroxidase activity. The slides were blocked with 10% goat serum. Then they were incubated with the antibodies overnight and followed by incubation with secondary antibodies. The chromogenic reaction was performed by DAB and the slides were then counterstained with hematoxylin. Expression quantification was analyzed using the IHC Profiler plugin in ImageJ. The analysis principle of the plugin is to calculate the color intensity (strong positive, positive, weak positive and negative) ratio (pi, ranging 1-100) in the selected area. Each color intensity (i) was determined as 3, 2, 1, and 0, respectively. Histological score (H-Score) was calculated using the formula as: H-Score = ∑pi(i+1).

### Statistics analysis

All data were statistically analyzed using GraphPad Prism 8.0, and the numerical variables were expressed as mean ± SD. Statistical tests were two-tailed unpaired *t*-test, One-way analysis of variance (ANOVA) and *P* < 0.05 was considered to be statistically significant.

### Study approvals

All animal experiments were approved by the animal welfare committee of the Fifth Affiliated Hospital of Sun Yat-sen University (2018120601).

## Results

### CDDP-activated JNK/c-Jun-ATF2 was related to chemoresistance in liver cancer cells

**Figure 1:**
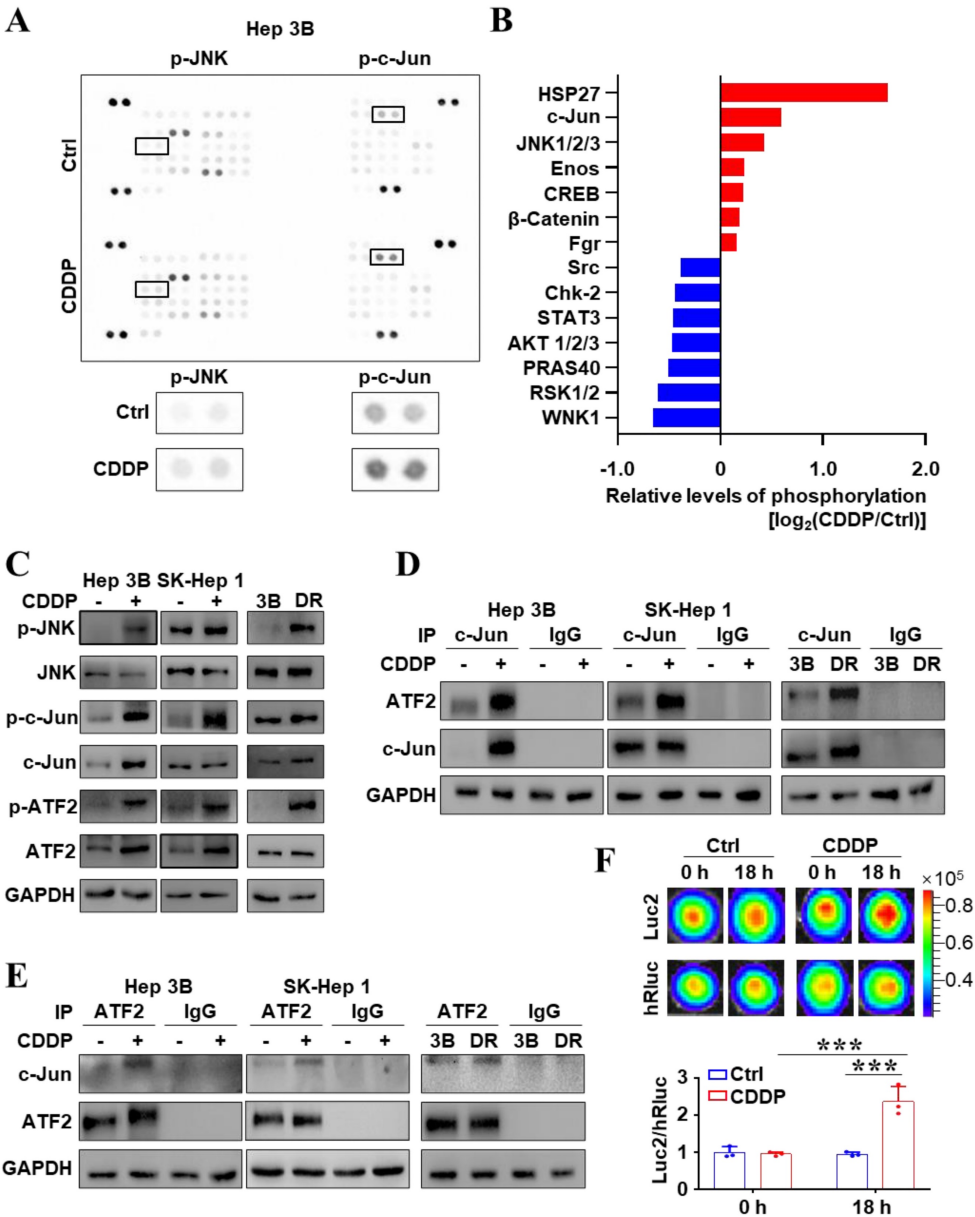
The activated JNK/c-Jun-ATF2 signaling was related to CDDP resistance in liver cancer cells. **(A, B)** Human phosphor-kinase array was performed after Hep 3B cells treated with CDDP (20 μM CDDP for 18 h) **(A)**. The quantification plot showed the relative levels of kinase phosphorylation sites **(B)**.** (C)** Western blot analysis illustrated the effect of CDDP (20 μM for 18 h) treatment on JNK signal pathway. The p-JNK, p-c-Jun and p-ATF2 were also detected in Hep 3B and Hep 3B/DR cells without CDDP treatment. **(D, E)** Co-IP assays were performed to detect the interaction between c-Jun and ATF2 upon CDDP treatment. Hep 3B and SK-Hep 1 cells were treated with 20 μM CDDP for 18 h. The interaction of c-Jun and ATF2 was also detected in Hep 3B and Hep 3B/DR cells without CDDP treatment. **(F)** SK-ATF-Luc cells were treated with CDDP (20 μM) for 18 h.* In vitro*, BLI detected the activity of Luc2 and hRluc. The quantification data were shown as the ratio of Luc2 and hRluc. ***P < 0.001.

The signal alteration induced by drugs is one of factors for tumors to evade treatment. Firstly, 37 different phosphorylation of human kinase phosphorylation sites were analyzed by the human phosphokinase array. Figure 1A, B showed that the phosphorylation level of JNK and c-Jun increased after CDDP treatment. JNK signaling transduction pathway is one of three well-characterized MAPK pathways regulating cellular physiological functions 19. c-Jun and ATF2, targets of JNK, usually constitute a heterodimer to mediate transcriptional regulation in many cell processes through binding to ATF element site of the promoter 18, 19. In order to identify the underlying mechanism of CDDP resistance, CDDP-resistant cell strain (Hep 3B/DR) was constructed by CDDP low-dose continuous stimulation (Figure S1A). And SK-Hep 1, more resistant to CDDP than Hep 3B, was also used here (Figure S1B). The western blot further affirmed that the levels of phosphorylation of JNK, c-Jun and ATF2 were significantly increased after CDDP treatment (Figure 1C). It is important to note that, the levels of p-JNK, p-c-Jun and p-ATF2 were also higher in Hep 3B/DR than those in the parent cell line Hep 3B (Figure 1C). More interestingly, the formation of c-Jun-ATF2 heterodimer increased when liver cancer cells were treated with CDDP (Figure 1D, E). Importantly, compared to Hep 3B, Hep 3B/DR had more c-Jun-ATF2 heterodimer (Figure 1D, E). The c-Jun-ATF2 heterodimer can enhance promoter activity through binding to ATF element. SK-Hep 1 cells stably expressed 2×ATF-Luc2-TK-hRluc (SK-ATF-Luc cells), in which Luc2 was driven by 2×ATF and hRluc was driven by TK promoter. Then, Figure 1F showed that the ATF activity (Luc2/hRluc) was increased after CDDP treatment in SK-ATF-Luc cells by *in vitro* BLI assay (*P* < 0.05). The dual-reporter gene assay also showed that CDDP treatment enhanced the ATF activity in liver cancer cells. More interestingly, Hep 3B/DR also displayed higher ATF activity than Hep 3B (*P* < 0.05) (Figure S1C). These data together indicated that the activated JNK/c-Jun-ATF2 signaling pathway was related to CDDP resistance in liver cancer cells.

### Inhibition of JNK/c-Jun-ATF2 reversed cisplatin resistance in liver cancer cells

**Figure 2:**
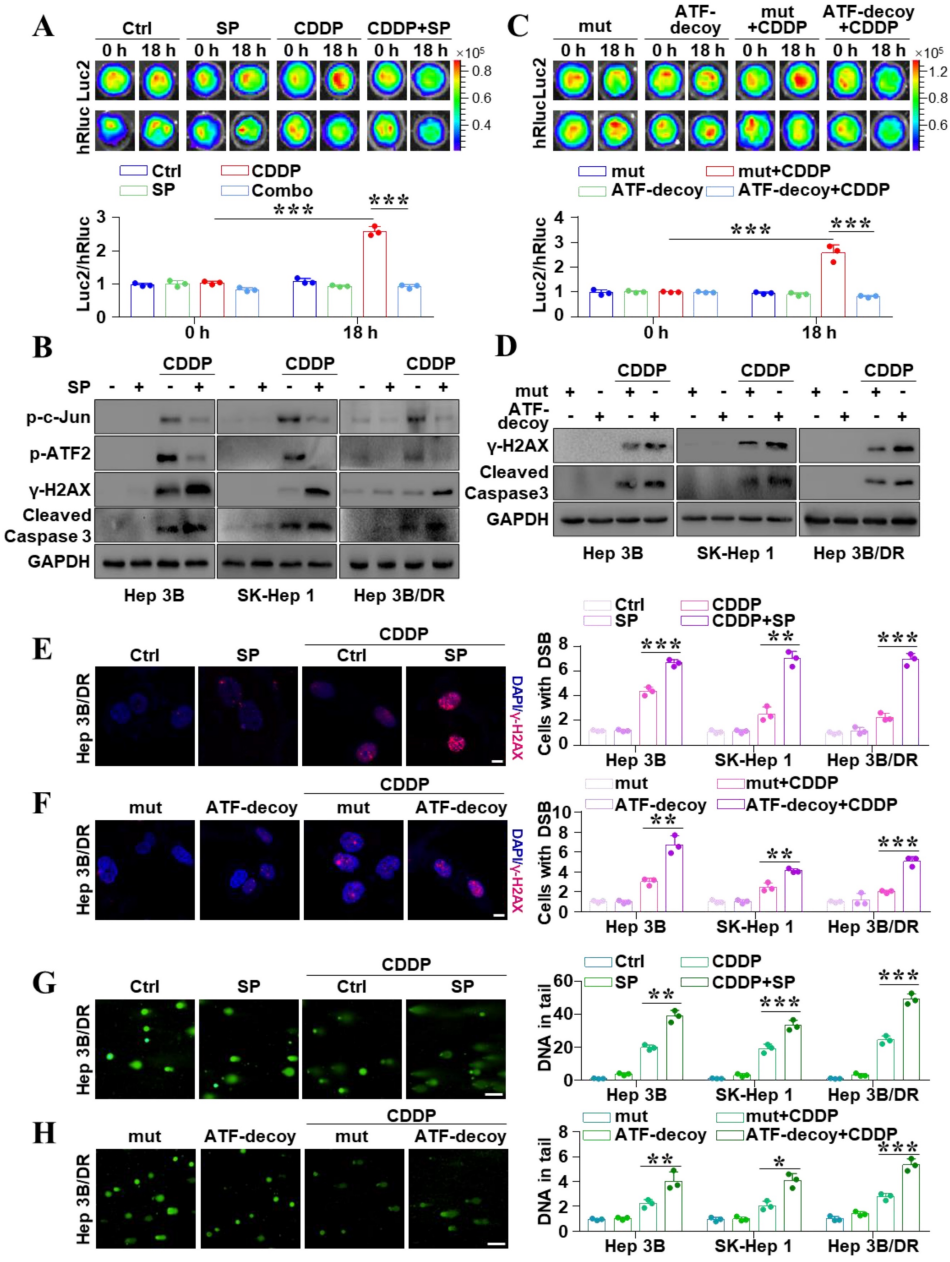
Inhibition of JNK/c-Jun-ATF2 reversed CDDP resistance in liver cancer cells. **(A)** SK-ATF-Luc cells were treated with SP600125 and CDDP (20 μM CDDP, 20 μM SP600125 for 18 h).* In vitro* BLI detected the activity of Luc2 and hRluc. The quantification data were shown as the ratio of Luc2 and hRluc. ***P < 0.001.** (B)** Western blot analysis illustrated the effect of SP600125, CDDP and the combination treatment effect on p-c-Jun, p-ATF2, γ-H2AX, and cleaved-caspase 3. **(C)** SK-ATF-Luc cells were transfected with ATF-decoy or mut-ATF-decoy (mut). *In vitro* BLI detected the activity of Luc2 and hRluc. The quantification data were shown as the ratio of Luc2 and hRluc. ***P < 0.001. **(D)** γ-H2AX and cleaved caspase 3 were assayed by western blot. **(E, F)** γ-H2AX was analyzed by IF in Hep 3B/DR cells. The representative pictures of Hep 3B and SK-Hep 1 were shown in Figure S3A and B. The nuclei were stained with DAPI. Scale bar, 10 μm. The quantification of the percentage of foci positive cells was shown. The data were presented as the mean ± SD of three different fields of view. **P < 0.01, ***P < 0.001. **(G, H)** Comet assay was performed for detecting DNA damage in Hep 3B/DR cells. The representative pictures of Hep 3B and SK-Hep 1 were shown in Figure S3C and D. The average tail moment per cell was quantified. Scale bar, 100 μm. *P < 0.05, **P < 0.01, ***P < 0.001.

To further explore whether the activation of JNK mediates drug resistance in liver cancer, JNK inhibitor SP600125 and ATF-decoy were used to inhibit JNK signal activity. *In vitro* BLI revealed that SP600125 weakened CDDP-induced activation of the ATF element (*P* < 0.05) (Figure 2A). And the dual-reporter gene analysis showed the same results (*P* < 0.05) (Figure S2A). Figure 2B also showed that SP600125 could remarkably reduce CDDP-induced high levels of p-c-Jun and p-ATF2. This data revealed that the activity of ATF element could reflect JNK activation. In addition, to determine the key role of c-Jun-ATF2 in CDDP-induced resistance, the ATF-decoy oligonucleotide with the consensus sequences of ATF was used to deplete c-Jun-ATF2, which disturbs them from binding to the ATF 20. Interestingly,* in vitro*, BLI showed that ATF-decoy partially abolished CDDP-induced activation of ATF (*P* < 0.05) (Figure 2C). And the dual-reporter gene analysis also displayed similar results (*P* < 0.05) (Figure S2B). Furthermore, SP600125 obviously increased the CDDP-induced upregulated γ-H2AX and cleaved-caspase 3 (Figure 2B). It is also noted that ATF-decoy rather than mut-ATF-decoy combined with CDDP promoted the DSBs and cell apoptosis (Figure 2D). Immunofluorescence confirmed that inhibition of JNK or disturbing the function of c-Jun-ATF2 obviously increased the CDDP-induced γ-H2AX (Figure 2E, F and Figure S3A, B) (*P* < 0.05). Moreover, the alkaline comet assay showed that SP600125 or ATF-decoy increased the CDDP-induced DSBs (*P* < 0.05) (Figure 2G, H and Figure S3C, D). These results suggested the activation of JNK/c-Jun-ATF2 by CDDP was associated with the resistance phenotypes of liver cancer cells. Collectively, inhibition of JNK/c-Jun-ATF2 reversed CDDP resistance in liver cancer cells.

### JNK/c-Jun-ATF2 facilitated resistance to CDDP by upregulating Galectin-1 in liver cancer cells

**Figure 3:**
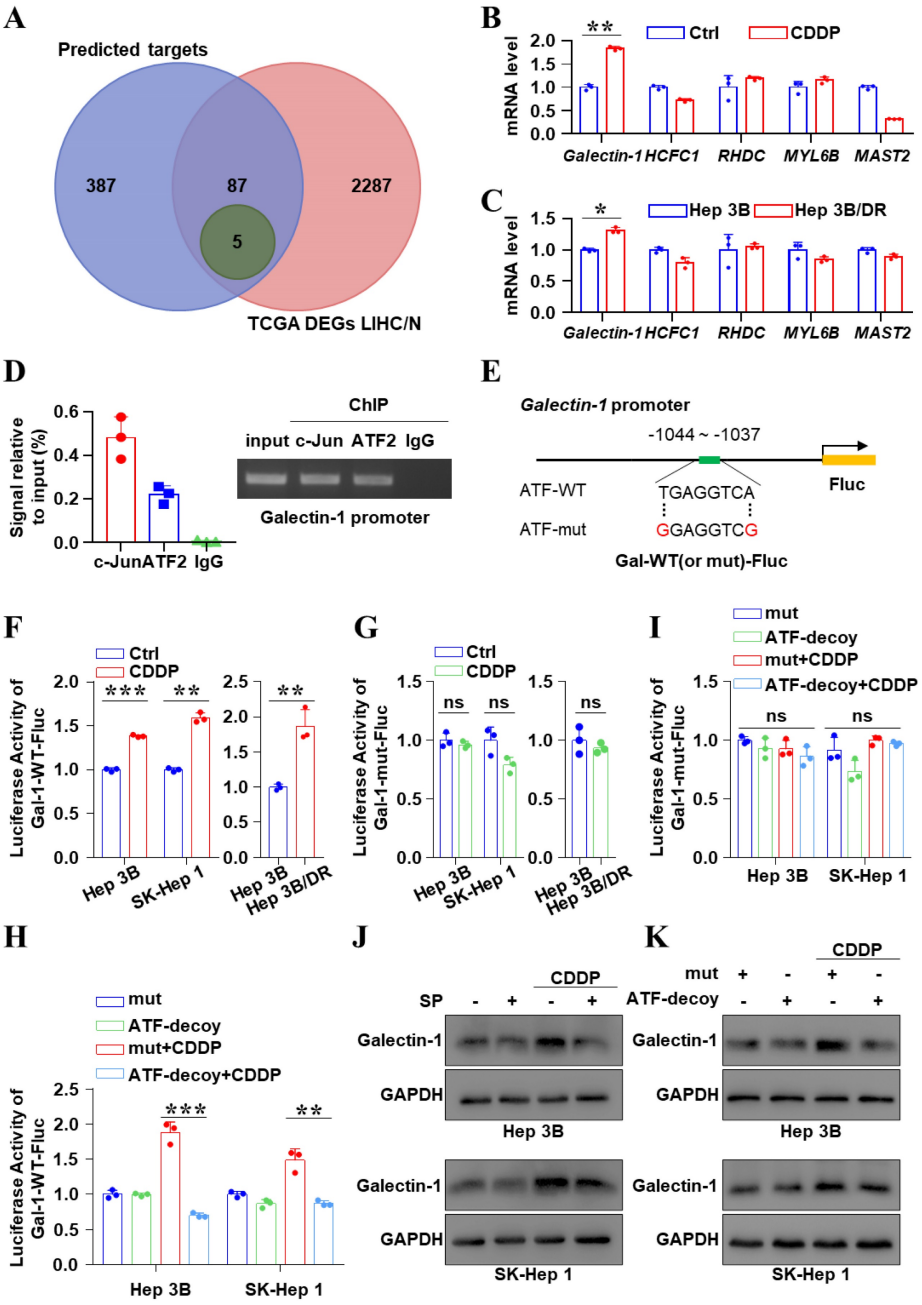
JNK/c-Jun-ATF2 upregulated the expression of Galectin-1 in liver cancer cells. **(A)** Venn diagram illustrating the number of genes enriched from three transcription factor databases and TCGA. LIHC (n = 371), N: normal (n = 50). The overlap (5 genes, green) indicates the number of genes found in all conditions. **(B, C)** Hep 3B cells were treated by CDDP (20 μM for 18 h) **(B)**. The quantification of mRNA for Galectin-1, HCFC1, RHDC, MYL6B and MAST2 was performed by qPCR. The levels of these genes were also detected in Hep 3B and Hep 3B/DR without CDDP treatment **(C)**. **(D)** ChIP-qPCR assays were performed using c-Jun and ATF2 antibodies in SK-Hep 1 cells (left). And the gel image was shown (right). **(E)** A schematic diagram showed the position of the c-Jun-ATF2 binding element (ATF) in human Galectin-1 promoter and sequence of the WT- (wild-type) and mut- (mutated) ATF in the luciferase reporter constructs. **(F, G)** Liver cancer cells were transfected with Gal-1-WT-Fluc or Gal-1-mut-Fluc, and were treated with CDDP (20 μM for 18 h). The dual-reporter analysis was performed. **P < 0.01, ***P < 0.001. **(H, I)** Liver cancer cells were transfected with ATF-decoy or mut-ATF-decoy (mut) and treated with CDDP (20 μM for 18 h). Dual-reporter assay was performed. **P < 0.01, ***P < 0.001. **(J)** Galectin-1 was assayed after SP600125 and CDDP treatment. **(K)** Western blot analysis of Galectin-1 after transfection with ATF-decoy.

Next, the downstream target of JNK/c-Jun-ATF2 was identified to further clarify the mechanism of CDDP resistance in liver cancer. A total of 92 genes overlapped between the targets of c-Jun or ATF2 and the DEGs analysis of liver cancer samples (Figure 3A). And among them, 59 genes are correlated with poor prognosis. Based on the JASPAR database, we finally affirmed 5 potential downstream targets: *Galectin-1*, *HCFC1*, *RHDC*, *MYL6B* and *MAST2* (Figure 3A-C). Interestingly, qPCR data showed that the *Galectin-1* mRNA increased in the CDDP-treated Hep 3B cells and was higher in Hep 3B/DR cells than that in Hep 3B (*P* < 0.05) (Figure 3B, C). A conserved putative ATF element was found in the *Galectin-1* promoter, located at -1037 to -1044. Importantly, ChIP assay showed c-Jun and ATF2 bound to *Galectin-1* promoter (Figure 3D). And two *Galectin-1*-promoter reporter genes Gal-1-WT and Gal-1-mut were constructed (Figure 3E). Dual-luciferase reporter assay revealed that CDDP treatment prominently enhanced the activity of *Galectin-1* promoter (*P* < 0.05). Its activity was also higher in Hep 3B/DR than in Hep 3B (*P* < 0.05) (Figure 3F). In contrast, the activity of Gal-1-mut-Fluc remained unchanged (Figure 3G). C2/c-Jun and C2/ATF2 are constitutively active mutants. The phosphorylation-dependent activation domain of the two mutants was replaced with the constitutively active transcriptional activation domain of the transcription factor CREB2. And as a result, the mutants could stimulate promoters containing ATF site 20. We found that Gal-1-WT-Fluc activity was increased after co-transfection of C2/c-Jun and C2/ATF2 (*P* < 0.05) (Figure S4A). In contrast, the activity of Gal-1-mut-Fluc was unchanged under the same condition (Figure S4B). In addition, the mRNA levels of Galectin-1 were significantly increased after co-transfection of C2/c-Jun and C2/ATF2 in liver cancer cells (Figure S4C). Moreover, ATF-decoy dramatically decreased the CDDP-induced activation of Gal-1-WT-Fluc (*P* < 0.05) (Figure 3H). But the activity of Gal-1-mut-Fluc remained unchanged (Figure 3I). Furthermore, qPCR and western blot showed that SP600125 and ATF decoy treatment decreased CDDP-induced Galectin-1 expression (Figure S5, Figure 3J, K and Figure S6). In general, JNK/c-Jun-ATF2 upregulated the expression of Galectin-1 in liver cancer cells.

### Inhibition of Galectin-1 reversed cisplatin resistance in liver cancer cells

**Figure 4:**
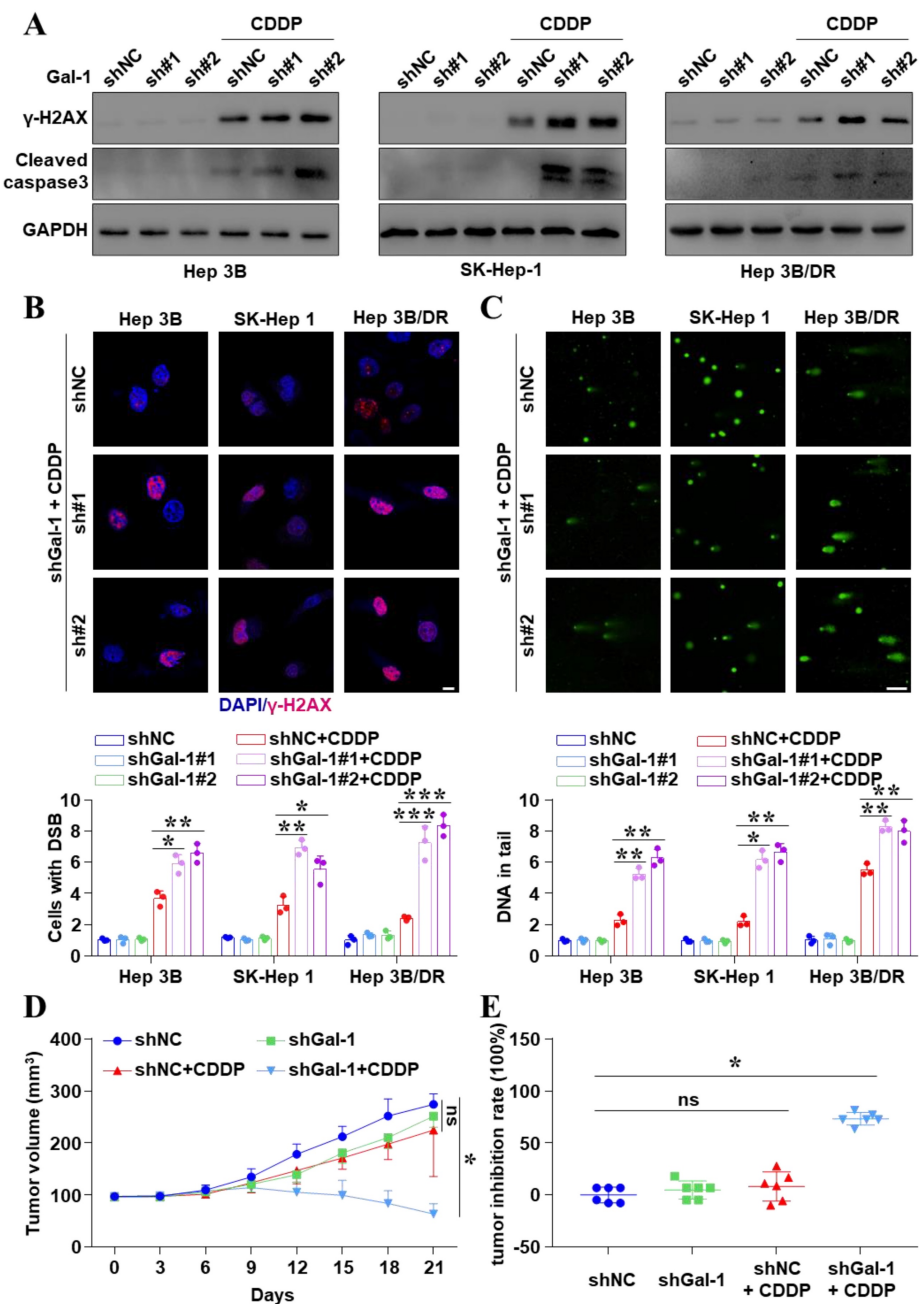
Galectin-1 knockdown reversed CDDP resistance in liver cancer cells. **(A)** Cells with Galectin-1 knockdown and CDDP treatment (20 μM for 18 h) were assayed for γ-H2AX and cleaved caspase 3 by western blot. **(B)** γ-H2AX was analyzed by IF. The nuclei were stained with DAPI. Scale bar, 10 μm. The quantification of the percentage of foci positive cells was shown. The data were presented as the mean ± SD of three different fields of view. *P < 0.05, **P < 0.01, ***P < 0.001. **(C)** Comet assay was performed for DNA damage, and the average tail moment per cell was quantified. Scale bar, 100 μm. *P < 0.05, **P < 0.01. **(D)** Galectin-1 has stably knocked down in SK-Hep 1 cells. Liver cancer cells-derived subcutaneous *xenograft*s were constructed. Animals were treated with CDDP (5 mg/kg). Tumor volumes were detected. The data were presented as the mean ± SD of six tumors. *P < 0.05. **(E)** Inhibition rate of tumor growth at the end of treatment. *P < 0.05.

Our findings confirmed that Galectin-1 was a downstream target of JNK/c-Jun-ATF2. And then, we investigated how Galectin-1 interfered with the therapeutic efficacy of CDDP treatment in liver cancer. Knocked down Galectin-1 in liver cancer cells (Figure S7) obviously increased the expression of the CDDP-induced γ-H2AX and cleaved caspase 3 (Figure 4A and Figure S8). IF assays also demonstrated that Galectin-1 knockdown significantly increased the number of CDDP-induced γ-H2AX foci (*P* < 0.05) (Figure 4B and Figure S9A). More interestingly, the comet assay showed that Galectin-1 knockdown significantly enhanced DSBs (*P* < 0.05) (Figure 4C and Figure S9B). We also found high expression of Galectin-1 increased the homologous recombination repair (HR) frequency (Figure S9C, D). Galectin-1 induced CDDP resistance by enhancing DDR. These basic findings were consistent with TCGA data showing that the higher level of Galectin-1 was related to poor clinical survival (Figure S10). Moreover, to explore the effect of Galectin-1 knockdown on CDDP efficacy* in vivo*, SK-Hep 1^shGalectin-1^ cell-derived *xenograft* models were constructed. Figure 4D demonstrated that CDDP alone could decrease the volume of tumors to some extent, but it did not make statistical sense. However, knockdown of Galectin-1 significantly enhanced the inhibitory effect of CDDP on tumor growth *in vivo* (*P* < 0.05). The inhibition rate of tumor was 73.45% in the combined group and 8.98% in the CDDP alone group (*P* < 0.05) (Figure 4E). In short, inhibition of Galectin-1 reversed CDDP resistance in liver cancer cells.

### The increasing JNK activity was monitored by *in vivo* real-time imaging after CDDP therapy in liver cancer

**Figure 5:**
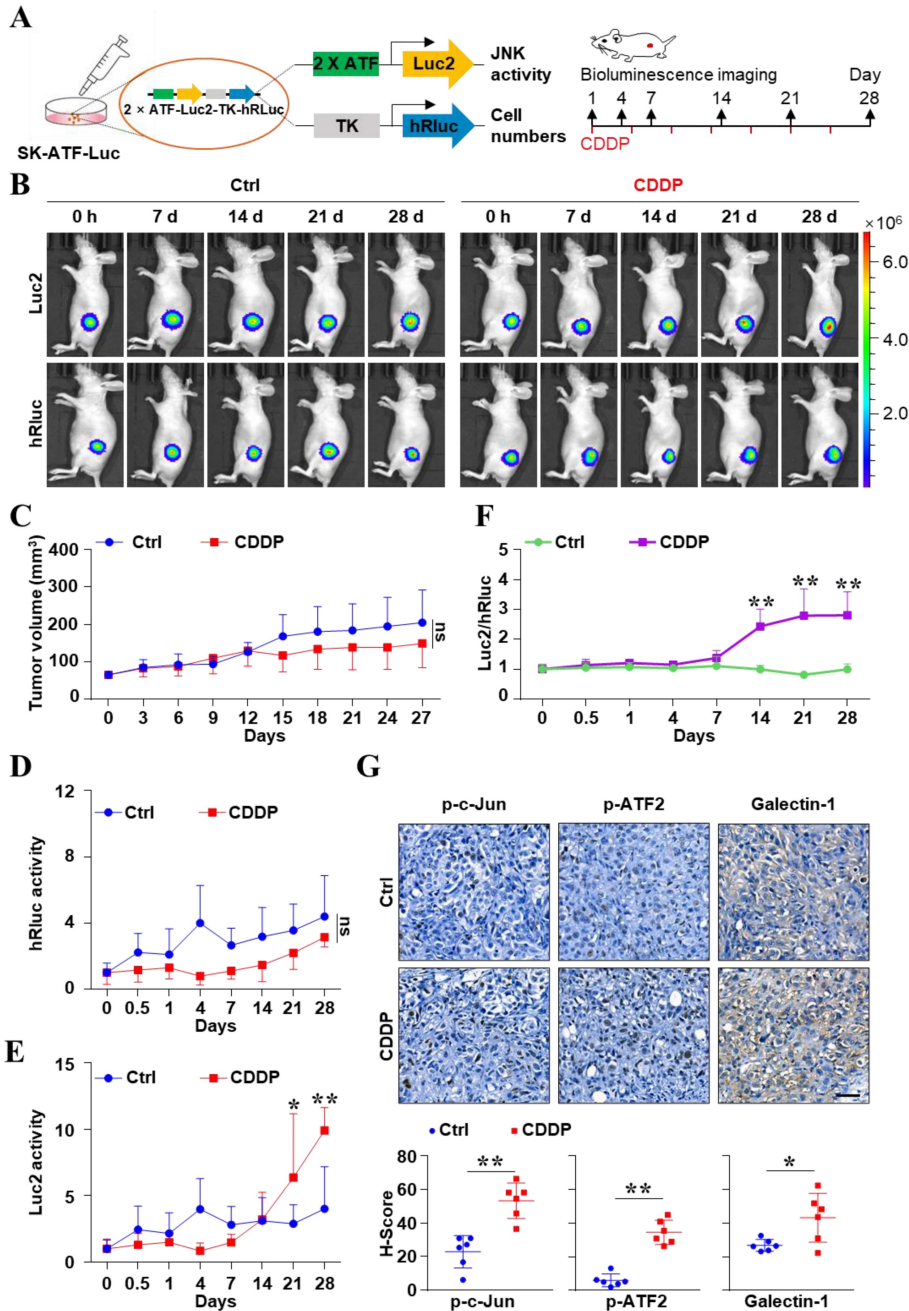
*In vivo* real-time imaging to monitor the activity of JNK/c-Jun-ATF2 during CDDP resistance evolution in liver cancer. **(A)** The *xenografts* were derived from SK-Hep 1 cells which were stably labeled with 2×ATF-Luc2-TK-hRluc. Experiment scheme illustrates when BLI and CDDP treatment were performed. **(B)** Representative bioluminescence images. **(C)** Mice were treated with vehicle or CDDP (5 mg/kg). Tumor size was monitored. ns: *P* > 0.05. **(D-F)** *In vivo*, BLI monitored the relative activity of ATF elements at the indicated times in PBS and CDDP groups. Quantification of the hRluc **(D)** or Luc2 (E) signals. And the ratio of Luc2 to hRluc (Luc2/ hRluc) was shown **(F)**. *P < 0.05, **P < 0.01. **(G)** Protein levels of p-c-Jun, p-ATF2 and Galectin-1 in resected tumors were analyzed by IHC. Scale bar, 50 μm. The data were presented as the mean ± SD of six tumors of view at low magnification (20×). *P < 0.05, **P < 0.01.

Based on the results at the cellular level, we proposed that the CDDP-activated JNK signal may attribute to the poor efficacy of CDDP alone for liver cancer. To investigate the dynamical change of JNK activity after CDDP treatment *in vivo*, we monitored the bioluminescent signal of JNK at the indicated times (Figure 5A). Figure 5B showed that the number of tumor cells and the absolute activity of ATF were obtained via humanized Renilla luciferase (hRluc) and humanized firefly luciferase (Luc2) imaging, respectively. CDDP could decrease the volume of tumors to some degree. But there was no statistical difference in tumor size between the PBS and CDDP groups (*P* > 0.05) (Figure 5C). And there was no difference in hRluc signal between the two groups (*P* > 0.0*5*) (Figure 5D). However, the mode for increasing Luc2 activity was different between the two groups. The activity of Luc2 in the CDDP group obviously increased from day 21 compared with the control (Figure 5E). This result meant the increase in absolute ATF activity. Notably, the relative activity of the ATF element (Luc2/hRluc) in the CDDP-treatment group significantly increased from day 14 and lasted until the 28th day (*P* < 0.05) (Figure 5F). But the activity of ATF element in the PBS treatment group remained unchanged (Figure 5F). IHC showed that the CDDP treatment increased p-c-Jun, p-ATF2 and Galectin-1 in tumor tissues (Figure 5G). These results showed that the activity of JNK abnormally increased after CDDP treatment *in vivo*, which reflected the acquisition of resistance in liver cancer.

### Inhibition of JNK reversed cisplatin resistance *in vivo* in liver cancer

**Figure 6:**
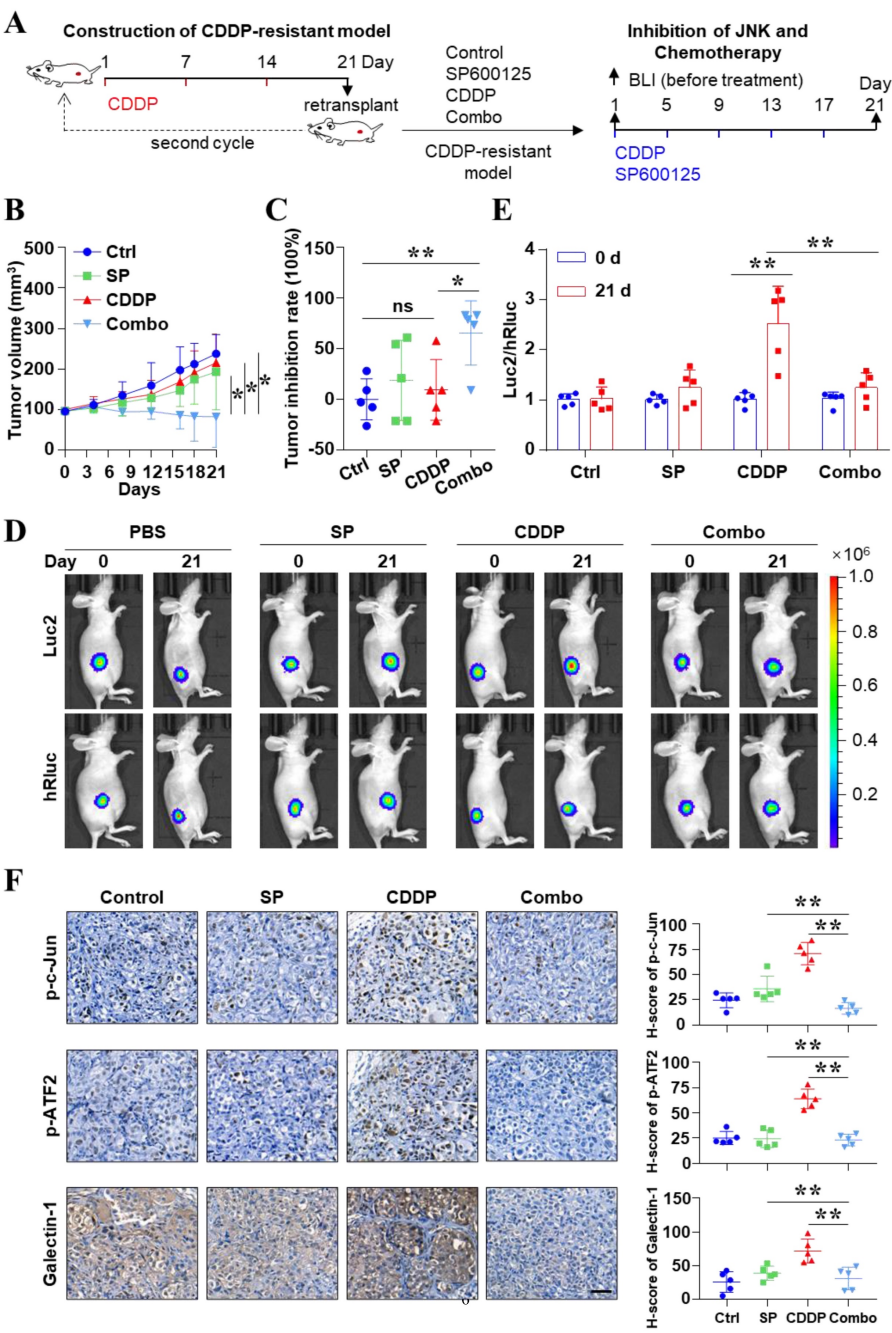
Inhibition of JNK reversed CDDP resistance *in vivo* in liver cancer*.* **(A)** Liver cancer *xenografts* were derived from SK-Hep 1 cells which were stably labeled with 2×ATF-Luc2-TK-hRluc. The schematic diagram shows the process of constructing the CDDP resistance model for liver cancer and the paradigm of treatment. **(B)** Effect of SP600125 (15 mg/kg), CDDP (5 mg/kg) and the combination of effects on tumor growth in *xenografts* mice. Tumor size was monitored. *P < 0.05. **(C)** Inhibition rate of tumor growth at the end of treatment with different formulations. *P < 0.05, **P < 0.01. **(D, E)** Representative bioluminescence images **(D)**. The ratio of Luc2 to hRluc (Luc2/ hRluc) was shown **(E)**. **P < 0.01. **(F)** Protein levels of p-c-Jun, p-ATF2 and Galectin-1 in resected tumors were analyzed by IHC. Scale bar, 50 μm. The data were presented as the mean ± SD of five different tumors of view at low magnification (20×). **P < 0.01.

Finally, CDDP-resistant animal models were used to further uncover the impact of high activity of JNK on CDDP efficacy (Figure 6A). These models were constructed by continuous treatment of relatively high doses of CDDP. Under such treatment conditions, some tumors decreased in size. The relatively large tumors were retransplanted to new mice. After two cycles of treatment and transplantation, the mice were considered as CDDP-resistant animal models (Figure 6A and see Materials and methods). Figure 6B showed that CDDP or SP600125 treatment alone could not inhibit tumor growth. But the combination of SP600125 and CDDP synergistically restrained tumor growth, although CDDP was used at the same low dose (*P* < 0.05). The inhibition rate was 65.79% in the combined group and 9.35% in the CDDP alone group (*P* < 0.05) (Figure 6C). More importantly, dual-luciferase BLI was performed at the end of treatment (Figure 6D). The Luc2/hRluc ratios of SP600125 treatment group were similar to those in PBS group. And the relative ATF activity significantly increased in CDDP group as before. Interestingly, the relative ATF activity remarkably decreased when mice were treated with SP600125 combined with CDDP (*P* < 0.05) (Figure 6E). Consistent with these biological effects, we found that CDDP treatment greatly enhanced the phosphorylation levels of c-Jun, ATF2, especially the level of Galectin-1. However, the p-c-Jun, p-ATF2 and Galectin-1 were suppressed in the combined treatment group (*P* < 0.05) (Figure 6F). In short, our data indicated that inhibition of JNK activity reversed CDDP resistance *in vivo* in liver cancer*.*

## Discussion

In this study, we demonstrated that the high activation of JNK was associated with CDDP resistance in liver cancer. JNK/c-Jun-ATF2 was found to mediate CDDP resistance through upregulating Galectin-1, which promoted DNA damage repair to enhance tumor CDDP resistance in liver cancer. For the first time, *in vivo* bioluminescence imaging (BLI) was used to dynamically monitor the acquisition of JNK high activation during the evolution of CDDP resistance. Inhibition of JNK activity reversed CDDP resistance* in vitro* and* in vivo*. Our findings revealed that JNK/c-Jun-ATF2/Galectin-1 is a potential target to reverse CDDP resistance in liver cancer.

JNK has been known as an important regulator to control adaptive responses to intracellular and extracellular stresses 24, 25. JNK has been found to mediate multiple cellular alterations via activating c-Jun, leading to cause the malignant progression of tumors 19. More and more evidence revealed that the activated JNK/c-Jun facilitates cervical cancer cell proliferation, promotes colon cancer metastasis and promotes breast cancer stem-like properties 26-28. In addition to c-Jun, ATF2 is also a common downstream protein of JNK, and the two proteins often form a heterodimer to act as a transcription factor 18. Our study found that CDDP activated JNK and enhanced the formation of c-Jun-ATF2 in liver cancer cells, while the function-depleted c-Jun-ATF2 increased CDDP treatment efficacy. Previous studies have reported that JNK signaling participates in CDDP resistance extensively among various tumors. The activation of JNK pathway was found to mediate CDDP resistance in endometrioid tumors 29. Similar results were also found in ovarian cancer, where platinum exposure led to JNK/c-Jun activation and further resulted in drug resistance 30. Furthermore, the inhibition of JNK/ATF2 sensitized non-small cell lung cancer to CDDP 31. These researches prompted us to further explore the molecular mechanism of CDDP resistance in liver cancer.

To the content of targets of c-Jun: ATF2, Hayakawa et al identified 269 genes as potential downstream targets of c-Jun and ATF2 in breast cancer cells 32. It is worth noting that CDDP treatment upregulates DNA damage repair-related genes, such as *ERCC1*, *XPA*, *RAD23B*, and *MSH2*. In our study, Galectin-1 was newly confirmed as the downstream target of c-Jun-ATF2 in liver cancer. Interestingly, Galectin-1 has not been identified as a target of c-Jun-ATF2 in the breast cancer cells mentioned above. As the prototype member belonging to the Galectin superfamily 33, Galectin-1 from the intercellular matrix has been found to promote the proliferation and metastasis of various tumors, such as lung, breast and ovarian cancer 34, 35. Recently, it was found that inhibition of Galectin-1 in tumor cells was related to elevated sensitivity to CDDP in lung cancer, neuroblastoma and epithelial ovarian cancer 36-38. Similarly, our findings indicated that inhibition of Galectin-1 enhanced the efficacy of CDDP. In liver cancer, Galectin-1 has been considered a bifunctional regulator of cell adhesion, polarization and growth 39. It was reported that the high level of Galectin-1 mediates doxorubicin resistance and metastasis 40, 41. Notably, we found out that the overexpression of Galectin-1 enhanced HR-mediated DDR to cause CDDP resistance. This finding may propose a new view on the impact of Galectin-1 in chemoresistance. In general, our work uncovered an essential role of CDDP-induced JNK/c-Jun-ATF2/Galectin-1 in chemoresistance.

In antitumor treatment, cancer cells trigger a cascade of signal changes through a dynamic evolution to survive 9. It remains elusive how and when these signaling switch. The real-time monitoring of malignant molecular activity enables the assessment to therapeutic response earlier and more precisely 6. Therefore, it is particularly important to explore when the kinase activity is changed and how to track this change *in vivo* during antitumor therapy. In monitoring JNK activity, a previous study detected its activation at the cellular level based on the fact that the phosphorylation state of a substrate peptide changes the signal of the ECFP/YFP fluorescence resonance energy transfer system 42. However, this method only suites to image at the cellular level instead of *in vivo* because of its phototoxicity and high background due to the requirement of excitation light 43. The principle of BLI is a chemical reaction between luciferase and luciferin, which produces a light signal. This technology has the advantages of high sensitivity, high specificity, and extremely low background due to the non-requirement of external excitation light 44. BLI-guided technology helps to achieve live visualization of the transformation of cell phenotypes in tumor tissues, enabling real-time assessment of therapeutic effect for treatment options 44, 45. Therefore, luciferase-based reporters are a better choice for monitoring JNK activity *in vivo*. We attempted to apply BLI to verify and explore the tumor response to therapy from the molecule perspective. Although researchers have used genetic engineering to try to optimize luciferases, the firefly/D-Luciferin system still performed excellently in imaging subcutaneous tumors 46. The Luc2 and hRluc used in our study were humanized from firefly luciferase and renilla luciferase, which were more suitable for expression in human tumor cells 47. In addition, the substrate of hRluc ViviRen used here is also suitable for *in vivo* imaging due to its less auto-oxidation, higher signal intensity and more stable than the natural substrate 47. Our cellular level data showed that the CDDP resistance was regulated by the high activity of JNK signaling. Therefore, we applied c-Jun-ATF2-responsive reporter system for dual BLI to achieve intravital tracing of JNK response in the context of CDDP treatment. In our study, we simulated the evolution of CDDP resistance in animals. Interestingly, the activity of JNK increased in the process of CDDP treatment* in vivo*, which reflected the acquisition of CDDP resistance. Due to the elevated JNK activity under drug resistance, we applied the JNK small molecule inhibitor in the CDDP resistance animal model. BLI revealed that combination therapy successfully halted the high activity of JNK and reversed CDDP resistance. In general, our study used *in vivo* BLI to quantitively track the process of CDDP resistance successfully. *In vivo* visualization of molecular events will contribute to the early discovery of tumor response to therapy and the development of measures to alleviate drug resistance.

In summary, this work confirmed that the high activity of JNK/c-Jun-ATF2/Galectin-1 mediates cisplatin resistance in liver cancer. Nevertheless, a few limitations should be mentioned. Some upstream molecules of JNK including MEKK4/MEKK7, TGF-β, etc., could be activated by intracellular and extracellular stress to affect JNK signal pathway in mediating drug resistance 48. Further explorations are needed to clarify the upstream molecules of JNK. Additionally, previous study found that Galectin-1 enhanced DDR in HeLa cells without determination of the repair type 49. While we found that Galectin-1 increased the frequency of HR-mediated DDR. But the molecular mechanism requires further investigation. Lastly, we used Luc2 and hRluc to track JNK signal activity in our study. Luc2 and hRluc are respectively emitted at 560 nm and 480 nm 44. And the emission wavelength close to the near-infrared window (650 nm-900 nm) is easy for deep tissue imaging 43. AkaLuc and Antares are emitted at 677 nm and 600 nm 50, 51. Therefore, we proposed that combining them would expand the technology of signal activity imaging to deep tissue.

## Conclusions

This study confirmed the essential role of the JNK/c-Jun-ATF2/Galectin-1 activated by cisplatin treatment itself in chemoresistance. Moreover, we constructed a bioluminescence reporter system 2×ATF-Luc2-TK-hRluc and clarified that it could reflect the activity of JNK signal. Furthermore, *in vivo* BLI revealed cisplatin gradually activated JNK signal activity during the evolution of chemoresistance. Inhibition of JNK decreased JNK activity and reversed drug resistance. In general, 2×ATF-Luc2-TK-hRluc is a better reporter system to monitor JNK activity *in vivo* and the monitoring of the malignant molecular alteration during therapy provides new prospects to explore the mechanism of drug resistance.

## Supplementary Material

Supplementary figures and tables.

## Figures and Tables

**Figure 7:**
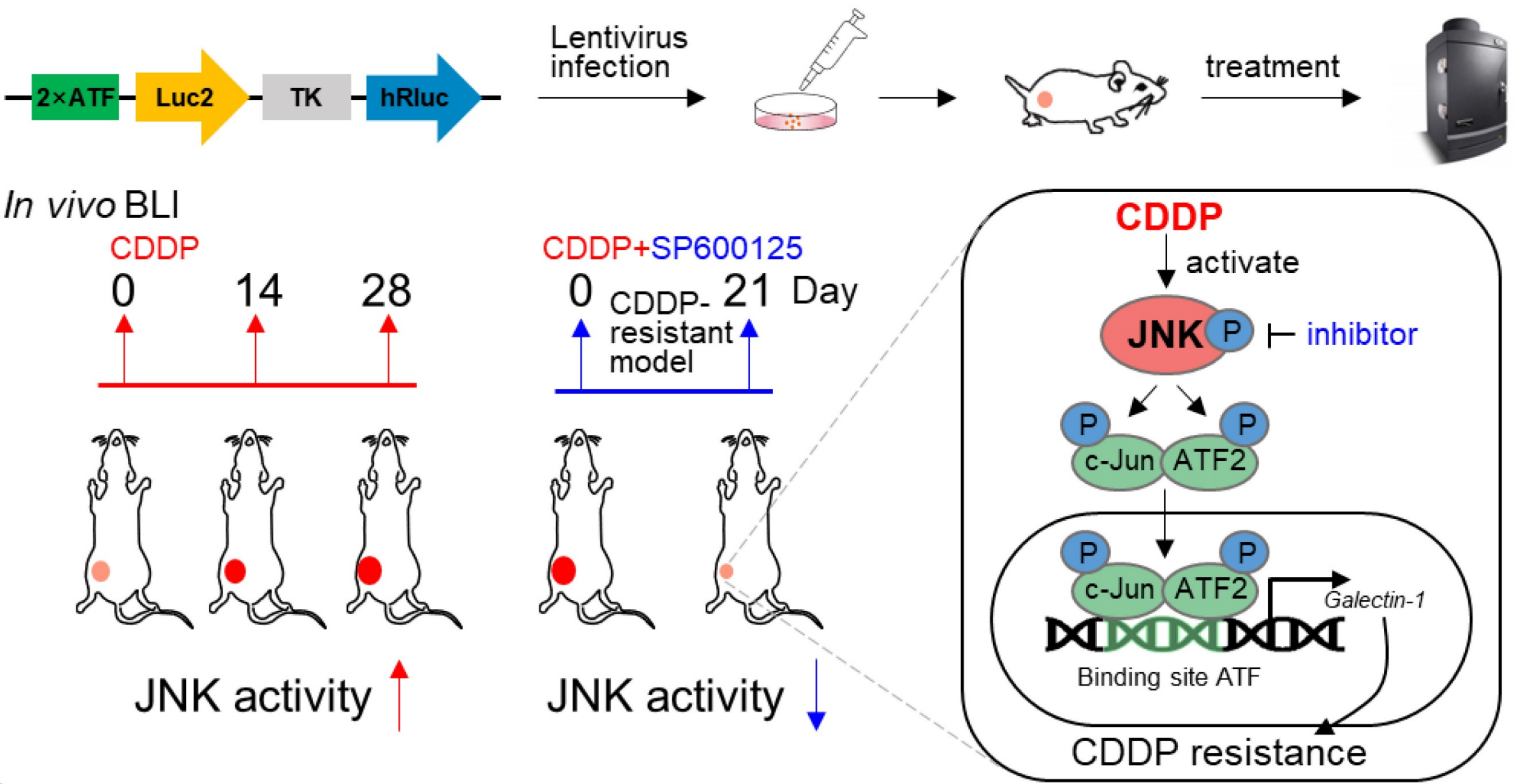
Diagram of *in vivo* monitoring JNK activity during JNK/c-Jun-ATF2/Galectin-1 mediated CDDP resistance.
