# Supplementary material for: Inhibition of JNK/c-Jun-ATF2 Overcomes Cisplatin Resistance in Liver Cancer through down-Regulating Galectin-1
Source: Int J Biol Sci. 2023 Apr 25;19(8):2366–81. doi: 10.7150/ijbs.79163 (PMC10197891; doi:10.7150/ijbs.79163)
Supplement: Supplementary file 1 — Supplementary figures and tables. [file ijbsv19p2366s1.pdf]

Supplementary information

Inhibition of JNK/c-Jun-ATF2 Overcomes Cisplatin Resistance in Liver Cancer through down-Regulating Galectin-1

Fan Yang[#], Mengzhu Li[#], Duo Xu[#], Zebo Jiang[#], Hailong Jiang, Yitai Xiao, Chaoming Mei, Meilin Yang, Congmin Chen, Bin Zhou, Bailiang He[*], Hong Shan[*], Pengfei Pang[*], Dan Li[*]

Figures S1-10

Tables S1-3

Methods S1-6

Supplementary Figures

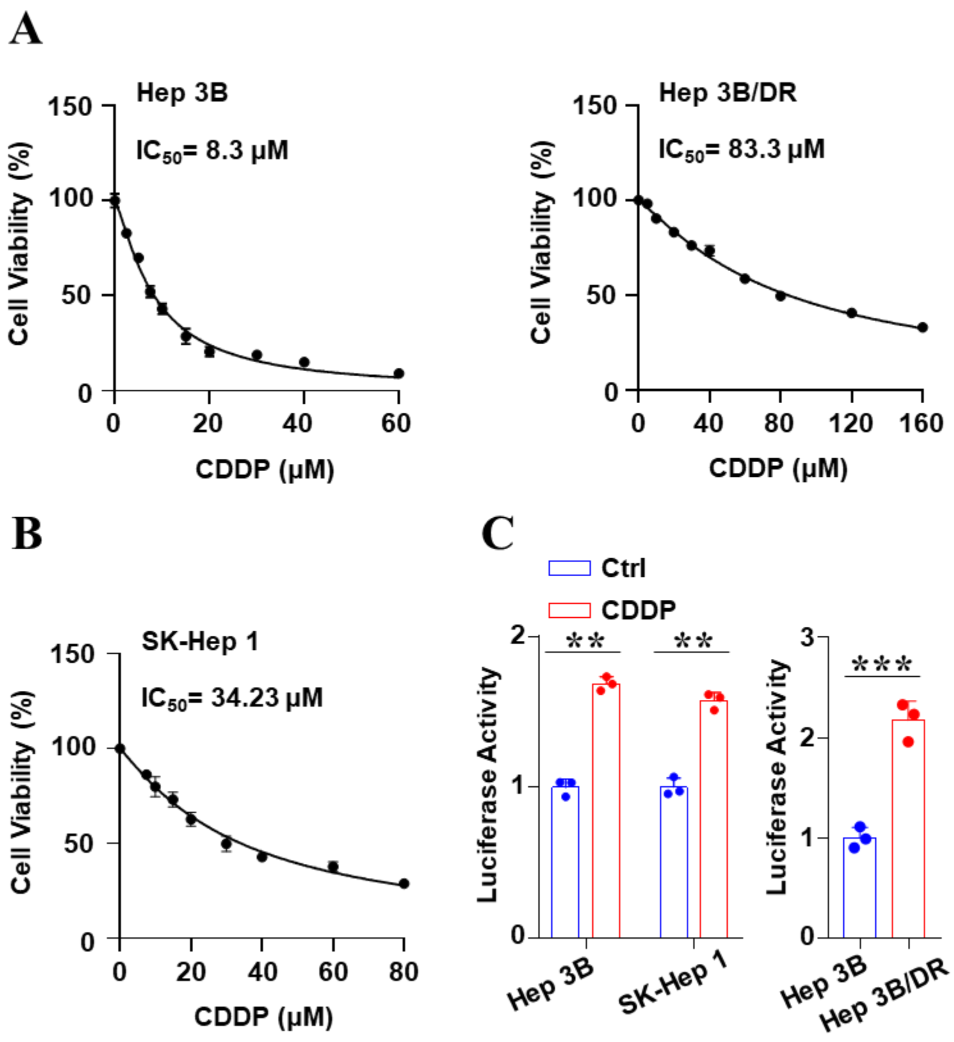

Figure S1. CDDP activated JNK signal in liver cancer cells. IC_{50} of CDDP was performed in Hep 3B, Hep 3B/DR (**A**) and SK-Hep 1 cells (**B**). (**C**) Dual-reporter assay detected the activity of ATF element. The levels of firefly luciferase activity were normalized to Renilla luciferase activity, which reflected the relative activity of ATF element. ***P* < 0.01, ****P* < 0.001.

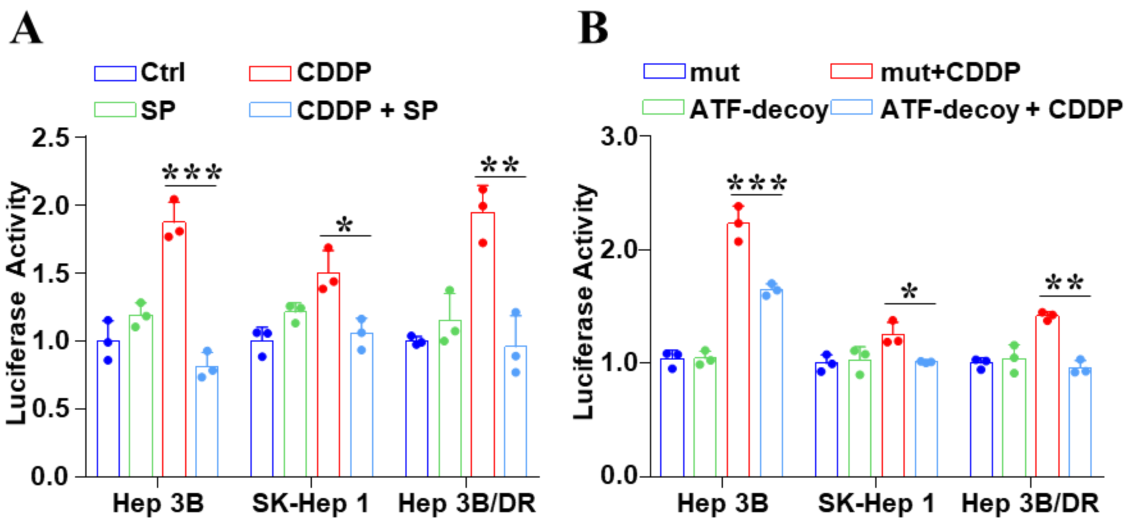

Figure S2. Inhibition of JNK/c-Jun-ATF2 by SP600125 or ATF-decoy decreased the activity of ATF element in liver cancer cells. (**A**) The relative activity of ATF elements was detected by dual-luciferase reporter assay after SP600125 and CDDP treatment. (20 μM CDDP, 20 μM SP600125 for 18 h) *$P < 0.05$, **$P < 0.01$, ***$P < 0.001$. (**B**) Cells were co-transfected with ATF-decoy or mut-ATF-decoy (mut) and pGL3-ATF-Fluc plasmids. The dual-reporter analysis was performed. *$P < 0.05$, **$P < 0.01$, ***$P < 0.001$.

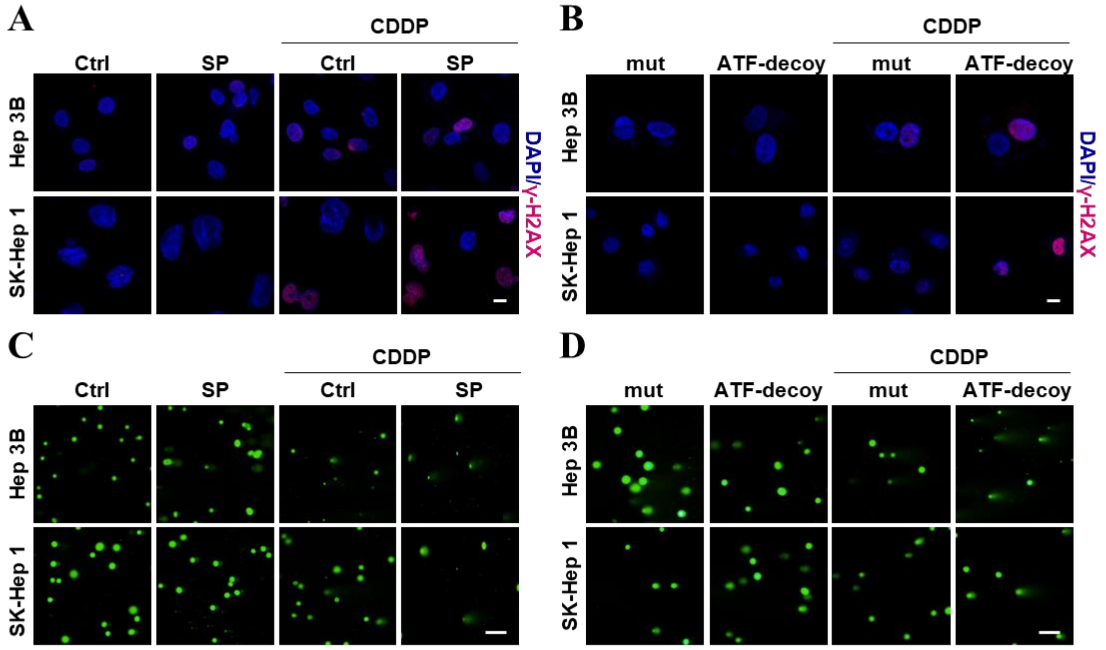

Figure S3. Inhibition of JNK/c-Jun-ATF2 by SP600125 or ATF-decoy enhanced DNA damage in liver cancer cells. (**A**, **B**) γ-H2AX was analyzed by IF in Hep 3B and SK-Hep 1 cells. The representative pictures were shown. The nuclei were stained with DAPI. Scale bar, 10 µm. The quantification of the percentage of foci positive cells was shown in Figure 2E, F. (**C**, **D**) Comet assay was performed for detecting DNA damage in Hep 3B and SK-Hep 1 cells. The representative pictures were shown. Scale bar, 100 µm. The quantification of the average tail moment per cell was shown in Figure 2G, H.

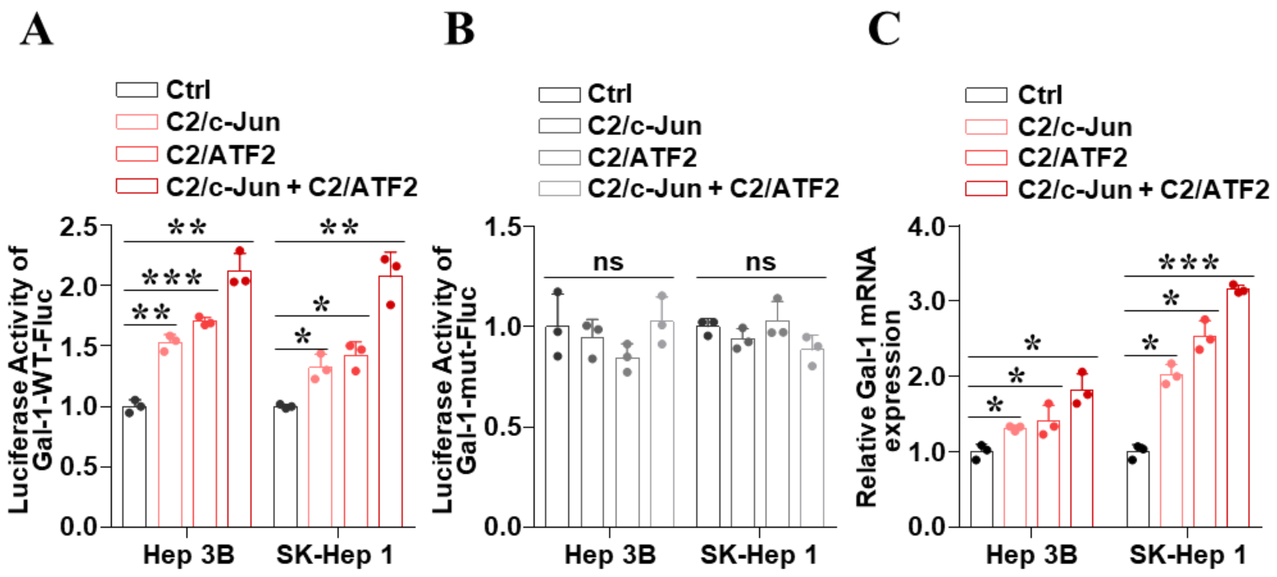

Figure S4. C2/c-Jun and C2/ATF2 enhanced the transcription of *Galectin-1* in liver cancer cells. (**A**, **B**) Cells were transfected with C2/c-Jun and C2/ATF2. The activity of Gal-1-WT-Fluc or Gal-1-mut-Fluc was measured by dual-reporter assay. (**C**) The *Galectin-1* mRNA levels were detected by qPCR. *$P < 0.05$, **$P < 0.01$, ***$P < 0.001$.

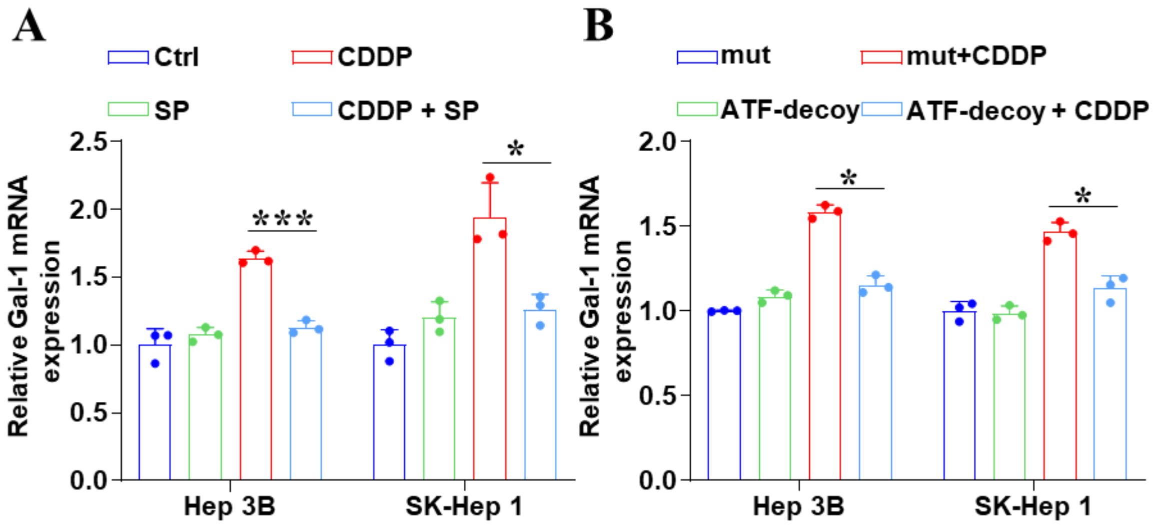

Figure S5. Inhibition of JNK/c-Jun-ATF2 by SP600125 or ATF-decoy decreased the mRNA level of *Galectin-1* in liver cancer cells. (**A**) Liver cells were treated with SP600125 and CDDP (20 µM CDDP, 20 µM SP600125 for 18 h). The *Galectin-1* mRNA levels were detected by qPCR. (**B**) Liver cancer cells were transfected with ATF-decoy or mut-ATF-decoy (mut) and treated with CDDP (20 µM for 18 h). The *Galectin-1* mRNA levels were detected by qPCR. *$P < 0.05$, **$P < 0.01$.

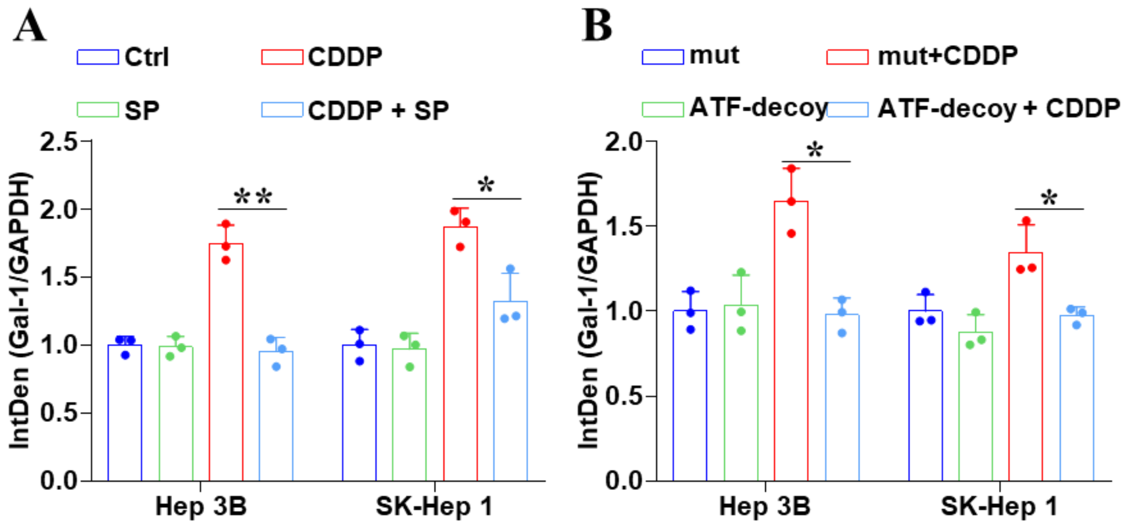

Figure S6. Inhibition of JNK/c-Jun-ATF2 by SP600125 or ATF-decoy decreased the protein expression of Galectin-1 in liver cancer cells. (**A**, **B**) The expression of Galectin-1 in western blot was quantitated by the gray density analysis. The representative images were shown in Figure 3J and K. *$P < 0.05$, **$P < 0.01$.

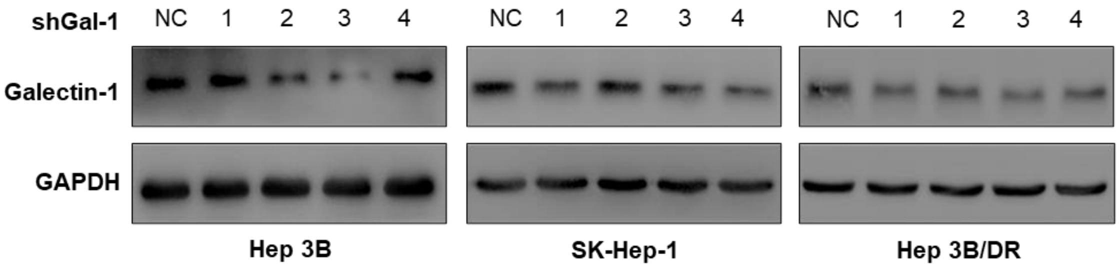

Figure S7. Galectin-1 was knocked down by shRNA. Liver cancer cells were transfected with sh Galectin-1. Galectin-1 protein was detected by western blot. A control shRNA and four Galectin-1-target shRNA were designed, represented as NC, 1, 2, 3 and 4 respectively.

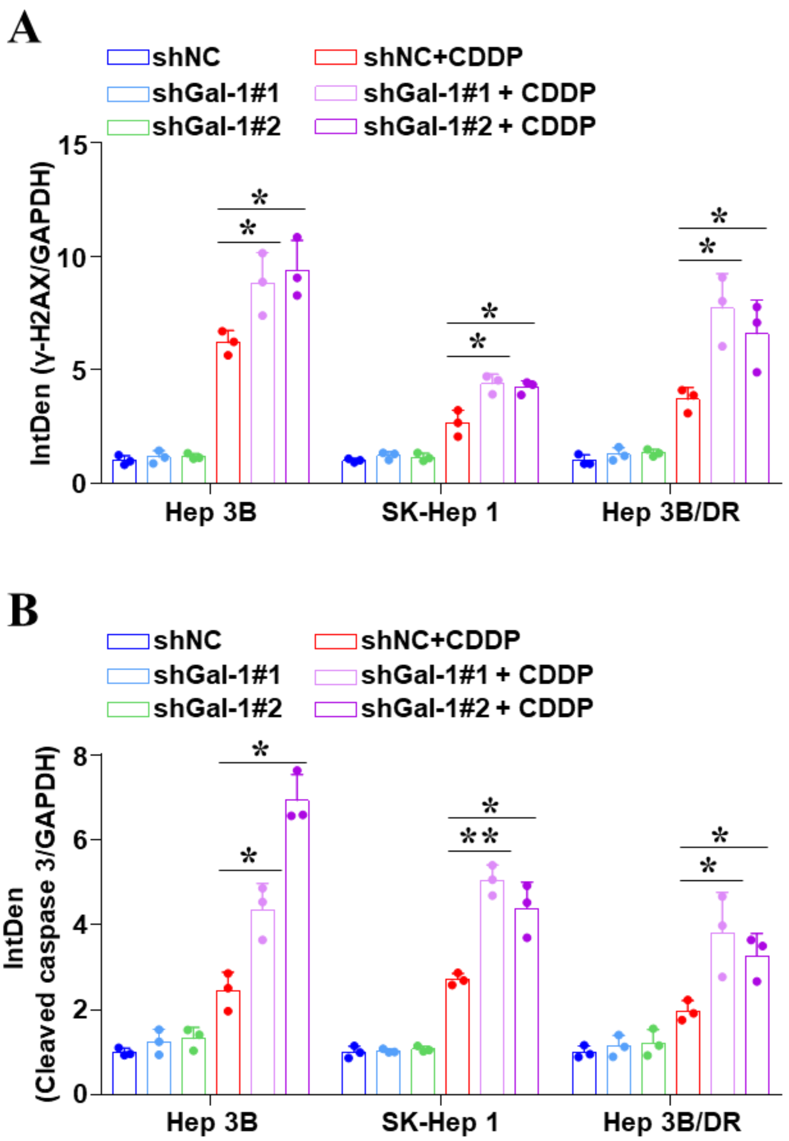

Figure S8. *Galectin-1* knockdown reversed CDDP resistance in liver cancer cells. (**A**, **B**) Cells with *Galectin-1* knockdown were treated with CDDP (20 μM for 18 h). The expression of γ-H2AX and cleaved caspase 3 was quantitated by the gray density analysis. The representative images were shown in Figure 4A. $^{*}P < 0.05$, $^{**}P < 0.01$.

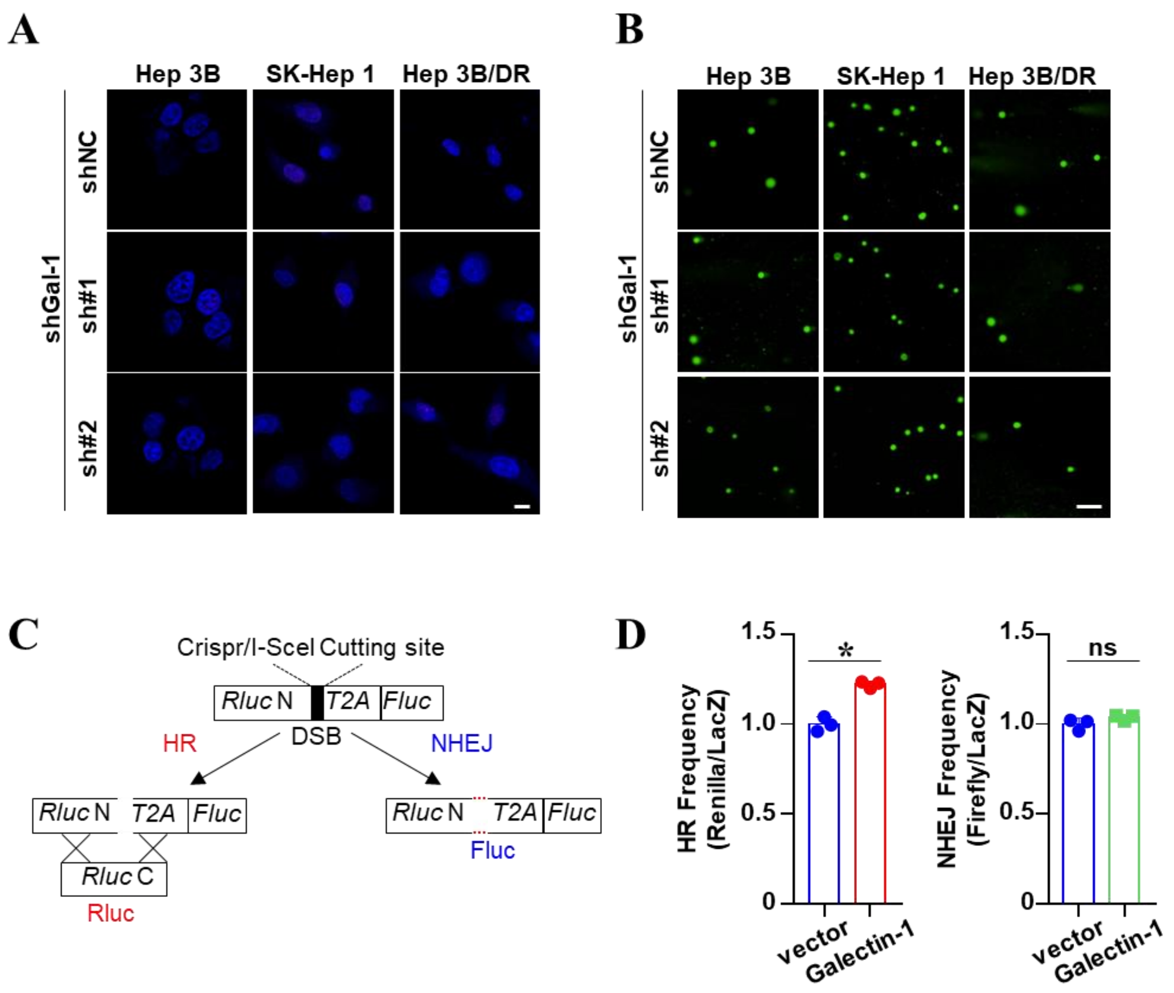

Figure S9. Galectin-1 hindered the efficacy of CDDP by mediating DNA homologous recombination repair. (**A**) Liver cancer cells were transfected with sh Galectin-1. γH2AX was analyzed by immunofluorescence. Cells were stained by γ-H2AX primary antibody and DAPI. The corresponding quantification results refer to figure 5B. Scale bar, 10 μm. (**B**) Liver cancer cells were transfected with sh Galectin-1. Comet assay was performed for detecting DNA damage. The corresponding quantification results refer to figure 5C. Scale bar, 100 μm. (**C**) The principle of determination of HR or NHEJ frequency. (**D**) Quantification of HR or NHEJ frequency in Galectin-1-overexpressing HEK293T cells.

A

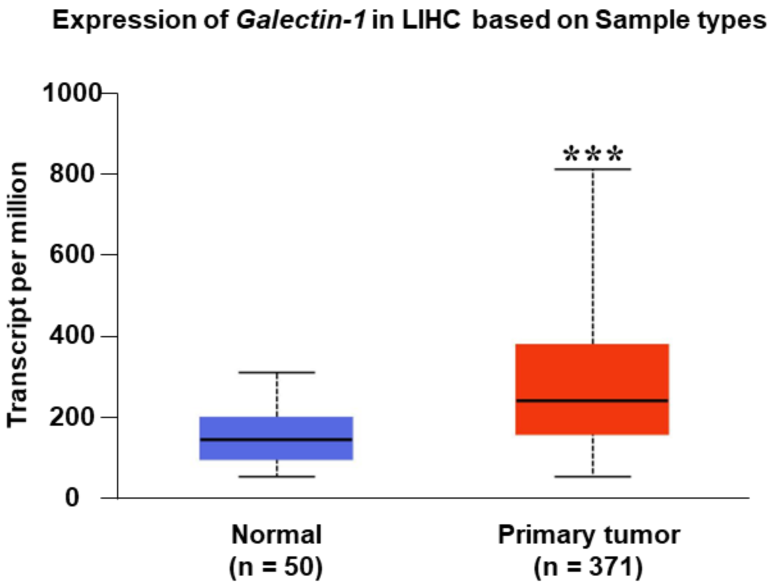

B

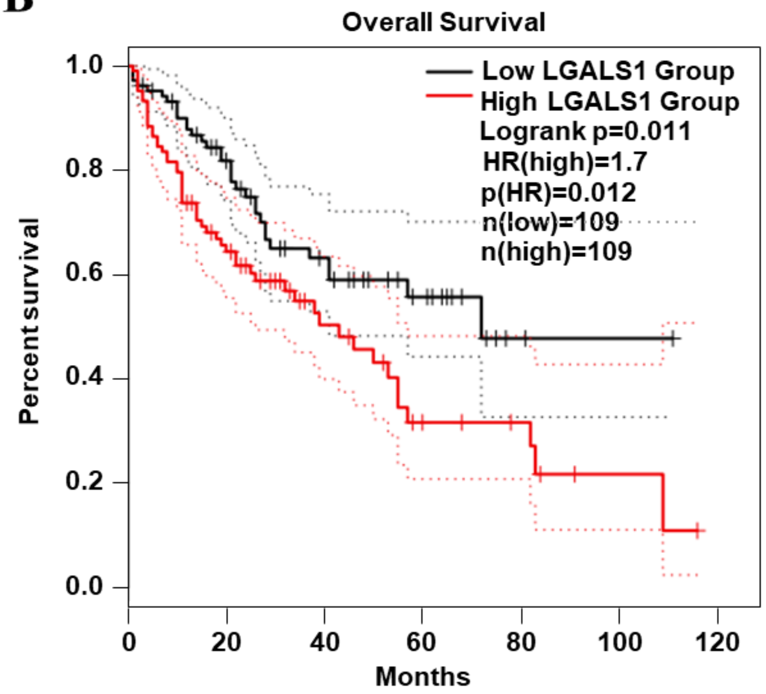

Figure S10. High expression of Galectin-1 is related to poor prognosis in liver cancer. (**A**) Expression profile of *Galectin-1* in liver cancer versus control tissues in TCGA dataset. (**B**) The overall survival analysis between high and low *Galectin-1* expression of liver cancer.

Supplementary Tables

Table S1

Name	Company	Catalogue
DMEM	Gibco	C11995500BT
RPMI-1640	Gibco	C11875500BT
fetal bovine serum	Gibco	10270-106
Cisplatin	Selleck Chemicals	S1166
SP600125	Selleck Chemicals	S1460
puromycin	Meilunbio	MA0318
BCA assay	Beyotime	P0010
Human Phospho-Kinase Array	R&D	ARY003C
RIPA	Beyotime	P0013C
protease inhibitor cocktail	Thermo Fisher	78430
loading buffer	EpiZyme	LT101S
PVDF membranes	BioTrace	66485
Dynabeads® Protein A	Invitrogen	1001D
Dual-Luciferase® Reporter Assay	Promega	P1910
BSA	Aladdin	A104912
Dylight 549-conjugated goat anti-rabbit IgG	Abbkine	A23320
DAPI	Solarbio	S2110
Alkaline comet	Trevigen	4250-050-K
SYBR Green I	Solarbio	SY1020
Total RNA Kit I	Omega	R6834-02
PrimeScript™ RT reagent Kit	Takara	RR047A
TB Green® Premix Ex TaqTM II	Takara	RR820A
ViviRen™	Promega	P1232
D-luciferin	PerkinElmer	122799
Galacto-Star™ System	Thermo Fisher	T1012
MaxVision™ HRP-Polymer anti-Rabbit IHC Kit	MXB Biotechnologies	KIT-5005
DAB Kit (20×)	MXB Biotechnologies	DAB-1031

Table S2

Antibody	Company	Catalogue	WB/IP/IHC/IF/ChIP	dilution
JNK	CST	9252S	WB	1:1000
c-Jun	CST	9165S	WB/IP/ChIP	1:1000/1:50/1:50
c-Jun	CST	2315S	WB	1:1000
ATF2	CST	35031S	WB/IP/ChIP	1:1000/1:50/1:50
p-JNK	CST	4668S	WB	1:1000
p-c-Jun	CST	3270S	WB/IHC	1:1000/1:200
p-ATF2	CST	9225S	WB/IHC	1:1000/1:200
γ-H2AX	CST	9718S	WB/IF	1:1000/1:200
Galectin-1	CST	12936S	WB	1:1000
Galectin-1	CST	13888	IHC	1:250
GAPDH	Proteintech	60004-1-Ig	WB	1:5000
rabbit IgG	CST	2729	ChIP	2 μL

Table S3

Name	Sequence
ATF-decoy	AATTCTTACCTCAGATGATTACCTCATCCCGCTTA-CCTCACGATCAGTTACCTCAC
mut-ATF-decoy	AATTCGGACCTCGGATGAGGACCTCGTCCCGCGGA-CCTCGCGATCAGGGACCTCGC
2×ATF	TCCCGCTTACCTCACGATCAGTTACCTCAC
MYL6B primers	forward 5’-GGAGCCTCCAGTCGATCTCT-3’<br>reverse 5’-GCAGGAAAGTCTCAAAGTCCAC-3’
RHOC primers	forward 5’-AGACCTGCCTCCTCATCGT-3’<br>reverse 5’-GGCCGCAGTCGATCATAGTC-3’
HCFC primers	forward 5’-ATCGAGTCCCTGGGTGTGA-3’<br>reverse 5’-GGTGCCCAAATCATCGTCTG-3’
Galectin-1 primers	forward 5’-TCGCCAGCAACCTGAATCTC-3’<br>reverse 5’-GCACGAAGCTCTTAGCGTCA-3’
MAST2 primers	forward 5’-ACGGAGGTGGTAGAGCTGAT-3’<br>reverse 5’-TAAGGGAAGAAAGGCTGCGG-3’
ACTB primers	forward 5’-GAGAAAATCTGGCACCACACC-3’<br>reverse 5’-GGATAGCACAGCCTGGATAGCAA-3’
Primers for ChIP-qPCR	forward 5’-TCAGAGGAGATGTTAAGAGAGCAGAC-3’<br>reverse 5’-CGTTGGCCAGGCTGGTC-3’

Supplementary methods

1. *CCK-8 assay IC_{50} of CDDP.*

For the determination of IC_{50} of CDDP in liver cancer cells, about 7000 cells/well were cultured in the 96-well plates. The cells were treated with CDDP at different concentrations for 24 h. CCK-8 reagent was diluted in the culture medium (10%). Each well was added 100 μL CCK-8 working solution and incubated in 37 °C incubators for 1.5 h. The absorbance of 450 nm was detected using Multimode Reader Synergy HTX (BIO-RAD).

2. *Plasmids.*

Plasmids pGL3-2×ATF-Fluc, pcDNA3.1-C2/c-Jun and pCMV-C2/ATF2 were gifts from Professor Mingtao Li (Zhongshan School of Medicine, Sun Yat-sen University). pLR-Renilla Luciferase (Rluc) was purchased from Promega.

3. *qPCR analysis*

Cells were transfected with ATF-decoy, mut-ATF-decoy, pcDNA3.1-C2/c-Jun or pCMV-C2/ATF2, and then treated or not treated with CDDP. RNA was obtained for qPCR assay. The relative expression of *Galectin-1* was analyzed according to the ΔΔCT relative quantification method and normalized to the expression of *ACTB*. Expression measurements of mRNA were performed in triplicate. The kit information was shown in Table S1. The primers are listed in Table S3.

4. *Dual-luciferase reporter assay*

pGL3-2×ATF-Fluc and pLR-Renilla plasmid were co-transfected to cells at ratio of 9:1 before drug treatment. To detect the effect of declined c-Jun-ATF2 on ATF element, cells were also co-transfected with the two plasmids above and ATF-decoy or mut-ATF-decoy. To determine the function of C2/c-Jun and C2/ATF2 on *Galectin-1* prompter, cells were co-transfected with Gal-1-WT-Fluc (or Gal-1-mut-Fluc) and pcDNA3.1-C2/c-Jun and pCMV-C2/ATF2. Dual-Luciferase® Reporter Assay was used to detect the activity of firefly and renilla luciferase.

5. *Determination of HR or NHEJ frequency.*

The plasmid system was a gift from Professor Yong Zhao (School of Life Sciences, Sun Yat-sen University, Guangzhou, China). This system was used to detect the frequency of homologous recombination (HR) or non-homologous end-joining (NHEJ) repair. The analysis was performed as the published paper [1, 2]. It consists

of three plasmids, including the DSB reporter plasmid, DSB repair templet plasmid and *LacZ* plasmid (transfection control). The three plasmids and pcDNA-Galectin-1 were co-transfected to HEK293T cells for 72 h. The luciferase activity was detected by Dual-Luciferase® Reporter Assay. The LacZ activity was detected by Galacto-Star™ System. The activity of firefly or renilla luciferase was normalized to LacZ activity.

6. Galectin-1 expression and survival analysis of liver cancer based on TCGA dataset.

The expression of *Galectin-1* mRNA in liver cancer was obtained from TCGA. The expression analysis and survival analysis were performed at http://ualcan.path.uab.edu/ and http://gepia2.cancer-pku.cn/#index.

1. Zhang C, Chen L, Peng D, et al. METTL3 and N6-Methyladenosine Promote Homologous Recombination-Mediated Repair of DSBs by Modulating DNA-RNA Hybrid Accumulation. Mol Cell. 2020; 79: 425-42 e7.
2. Xie C, Chen YL, Wang DF, et al. SgRNA Expression of CRIPSR-Cas9 System Based on MiRNA Polycistrons as a Versatile Tool to Manipulate Multiple and Tissue-Specific Genome Editing. Sci Rep. 2017; 7: 5795.
